# One Carbon Metabolism in SAR11 Pelagic Marine Bacteria

**DOI:** 10.1371/journal.pone.0023973

**Published:** 2011-08-23

**Authors:** Jing Sun, Laura Steindler, J. Cameron Thrash, Kimberly H. Halsey, Daniel P. Smith, Amy E. Carter, Zachary C. Landry, Stephen J. Giovannoni

**Affiliations:** 1 Department of Microbiology, Oregon State University, Corvallis, Oregon, United States of America; 2 Department of Botany and Plant Pathology, Oregon State University, Corvallis, Oregon, United States of America; Argonne National Laboratory, United States of America

## Abstract

The SAR11 *Alphaproteobacteria* are the most abundant heterotrophs in the oceans and are believed to play a major role in mineralizing marine dissolved organic carbon. Their genomes are among the smallest known for free-living heterotrophic cells, raising questions about how they successfully utilize complex organic matter with a limited metabolic repertoire. Here we show that conserved genes in SAR11 subgroup Ia (*Candidatus* Pelagibacter ubique) genomes encode pathways for the oxidation of a variety of one-carbon compounds and methyl functional groups from methylated compounds. These pathways were predicted to produce energy by tetrahydrofolate (THF)-mediated oxidation, but not to support the net assimilation of biomass from C1 compounds. Measurements of cellular ATP content and the oxidation of ^14^C-labeled compounds to ^14^CO_2_ indicated that methanol, formaldehyde, methylamine, and methyl groups from glycine betaine (GBT), trimethylamine (TMA), trimethylamine N-oxide (TMAO), and dimethylsulfoniopropionate (DMSP) were oxidized by axenic cultures of the SAR11 strain *Ca.* P. ubique HTCC1062. Analyses of metagenomic data showed that genes for C1 metabolism occur at a high frequency in natural SAR11 populations. In short term incubations, natural communities of Sargasso Sea microbial plankton expressed a potential for the oxidation of ^14^C-labeled formate, formaldehyde, methanol and TMAO that was similar to cultured SAR11 cells and, like cultured SAR11 cells, incorporated a much larger percentage of pyruvate and glucose (27–35%) than of C1 compounds (2–6%) into biomass. Collectively, these genomic, cellular and environmental data show a surprising capacity for demethylation and C1 oxidation in SAR11 cultures and in natural microbial communities dominated by SAR11, and support the conclusion that C1 oxidation might be a significant conduit by which dissolved organic carbon is recycled to CO_2_ in the upper ocean.

## Introduction

C1 metabolism takes place through a network of interrelated biochemical reactions that involves the transfer of one-carbon units from one compound to another. C1 units can be donated in the form of methyl (-CH_3_), methylene (-CH_2_-), methenyl (-CH = ), formyl (-CHO) and formimino (-CH = NH) groups [1], [2]. A few specialized bacteria oxidize methyl groups and C1 compounds, such as methanol, formaldehyde, formate and methylamine, to derive energy and cellular carbon. The most well known of these organisms are methylotrophs, which assimilate C1 carbon into biomass via the ribulose monophosphate (RuMP) or serine cycle pathways [3]–[7]. Less well known are organisms that have C1 oxidation pathways for energy production, but lack pathways for the net synthesis of biomass from C1 precursors [8].

Marine dissolved organic carbon (DOC) includes a diverse array of C1 and methylated compounds that are potential substrates for C1 oxidation. The most common methylated compounds in marine environments are osmolytes such as GBT, TMAO, and DMSP [9]–[11]. Methanol is a major component of oxygenated volatile organic chemicals in the oceans and atmosphere [12], [13]. Air measurements over the Pacific Ocean indicate that sea surface methanol concentrations are about 100 nM and that central ocean regions are net sinks for methanol deposited from the atmosphere [12]–[14]. Formaldehyde is ubiquitous in seawater. Likely sources of seawater formaldehyde are atmospheric deposition from industrial emissions and the photo-oxidation of atmospheric hydrocarbons [15], [16], and the photo-oxidation of dissolved organic carbon in the ocean surface [17]. The metabolism of methylated compounds in mammals also produces formaldehyde [18]. Formaldehyde is a key reactive intermediate in bacterial metabolism of C1 growth substrates like methane or methanol [19]-[21], and it is also a central intermediate of GBT methyl group oxidation [22]. Due to its nonspecific reactivity with proteins and DNA, formaldehyde is toxic to cells, and thus many studies have examined mechanisms by which organisms can remove this potentially lethal compound [23], [24].

Marine bacteria of the SAR11 clade are the most abundant aerobic, free-living, heterotrophic bacteria in ocean surface waters [25], [26]. SAR11 was first discovered in the Sargasso Sea in 1990 [27] and is now considered to be one of the most successful organisms on the planet. *Candidatus* Pelagibacter ubique strain HTCC1062, the first cultured SAR11 strain, has one of the most compact genomes known for a free-living organism (1,308,759 base pairs), and has dispensed with many functions that are common in other free-living bacteria, apparently in response to selective pressure for small genome size [28]. The SAR11 clade is divided into subclades that have distinct temporal and spatial distributions in the environment that are believed to represent ecotypes or species [29]. Most strains now being studied in laboratories belong to the Group Ia subclade, which is common in the ocean euphotic zone [29].

In this study, we observed a suite of genes for demethylation and C1 oxidation in three SAR11 Ia genomes (*Ca.* P. ubique strains HTCC1062, HTCC1002, and HTCC7211) for which no role in SAR11 had yet been identified. Genomic and metagenomic data were used to explore the potential for C1 metabolism in the three SAR11 isolates and natural SAR11 populations, and experiments with pure cultures of cells were used to confirm predictions. Together, our data demonstrate that SAR11 cells are capable of oxidizing a range of C1 compounds and methyl groups from methylated compounds to produce energy.

## Results and Discussion

### Comparative Genome Analyses and Proposed Metabolic Pathways

**Figure 1:**
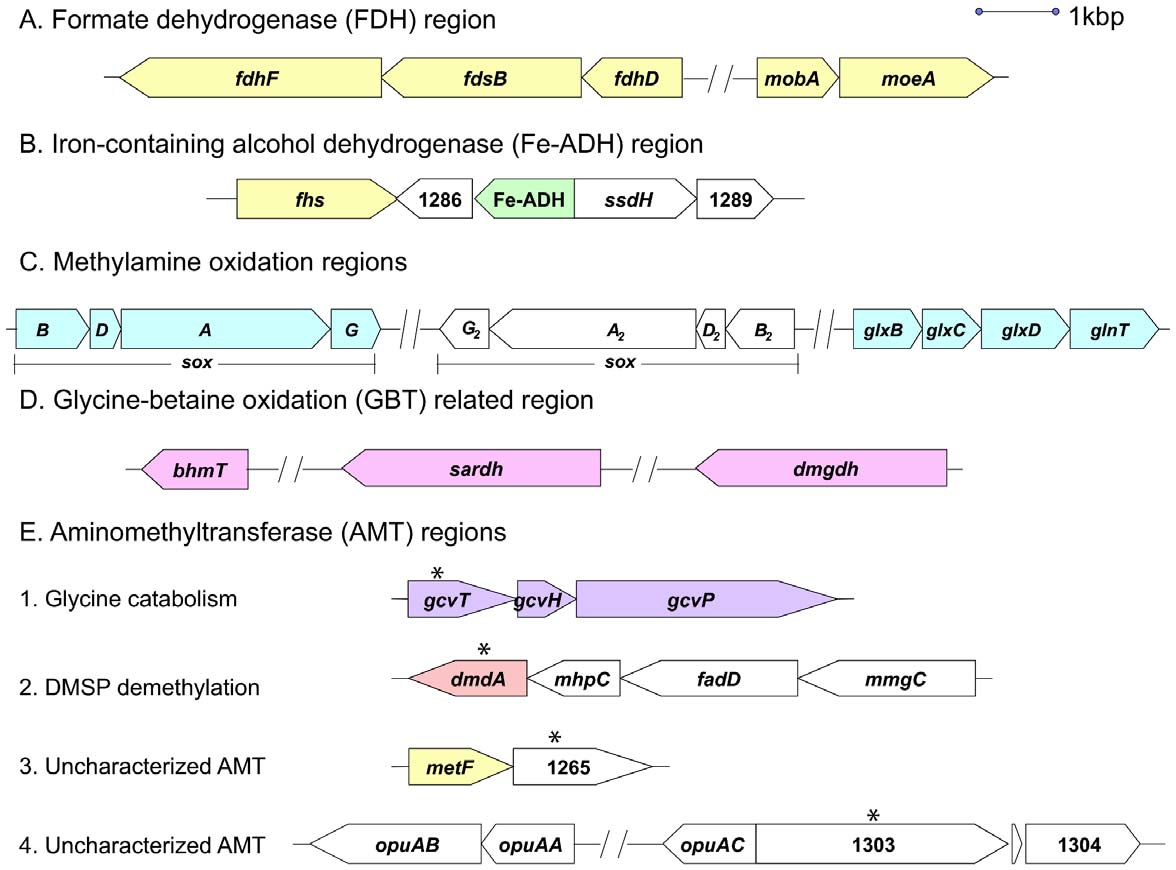
Demethylation and C1 oxidation regions of the strain HTCC1062 genome. (A) formate dehydrogenase; (B) methanol metabolism; (C) methylamine oxidation; (D) glycine betaine oxidation; (E) aminomethyltransferases (Asterisk). ***fdhF***, formate dehydrogenase, alpha subunit; ***fdsB***, NAD-dependent formate dehydrogenase, beta subunit; ***fdhD*** *,* formate dehydrogenase, chain D; ***mobA*** *,* molybdopterin-guanine dinucleotide biosynthesis protein A; ***moeA***, molybdopterin biosynthesis protein; ***fhs*** *,* formate-THF ligase; SAR11_1286, putative glutamine amidotransferase; **Fe-ADH,** iron-containing alcohol dehydrogenase; ***ssdH,*** aldehyde dehydrogenase family; SAR11_1289, short chain dehydrogenase; ***soxB***, sarcosine oxidase; ***soxD*** & ***soxD_2_***, sarcosine oxidase delta chain; ***soxA*** & ***soxA_2_***, sarcosine oxidase alpha chain; ***soxG*** & ***soxG_2_***, sarcosine oxidase gamma subunit; ***soxB***_2_, sarcosine oxidase beta subunit; ***glxBCD***, glutamate synthase; ***glnT***, Glutamine synthetase III (putative gamma-glutamylmethylamide synthetase); ***bhmT***, betaine-homocysteine methyltransferase; ***sardh***, sarcosine dehydrogenase; ***dmgdh***, dimethylglycine dehydrogenase; ***gcvT*** *,* glycine system cleavage T-protein; ***gcvH*** *,* glycine cleavage H-protein; ***gcvP*** *,* glycine cleavage P-protein; ***dmdA*** *,* dimethylsulfoniopropionate-dependent demethylase; ***mhpC***, hydrolase, alpha/beta hydrolase fold family; ***fadD***, CoA activator for DMSP beta oxidation; ***mmgC*** *,* acyl-CoA dehydrogenase for DMSP beta oxidation; ***metF,*** methylene-THF reductase; ***opuAB*** *,* glycine betaine transport system permease protein; ***opuAA*** *,* glycine betaine transport ATP-binding protein; ***opuAC*** *,* substrate-binding region of ABC-type glycine betaine transport system; SAR11_1265 & SAR11_1303, gcvT-like aminomethyltransferase protein; SAR11_1304, monomeric sarcosine oxidase. Colors correspond to pathways in Figure 2.

**Figure 2:**
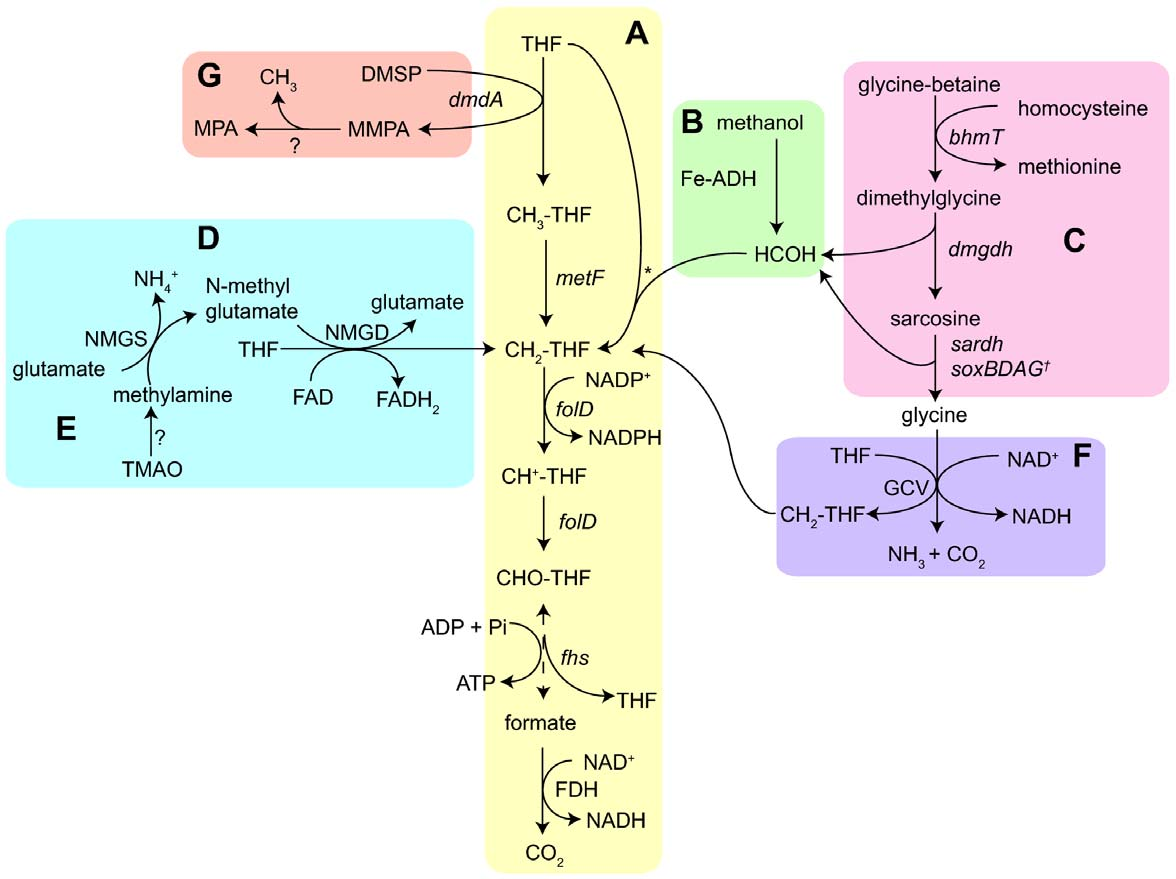
Proposed C1 and methylated compound oxidation pathways in SAR11 Group Ia. (A) THF-linked oxidation pathway; (B) methanol oxidation pathway; (C) glycine betaine demethylation and oxidation; (D) methylamine oxidation pathways; (E) TMAO degradation pathway; (F) glycine cleavage pathway; (G) DMSP demethylation. Note: **?** - unidentified metabolic processes/enzymes; * - spontaneous reaction; **†** - two paralogous operons.

Genes in the HTCC1062 genome associated with C1 oxidation are shown in Figure 1. The proposed metabolic pathways for these genes are discussed in detail below (Fig. 2), although for clarity we include only the HTCC1062 gene identifiers (e.g, “SAR11_”) in the text. Table S1 lists C1 metabolism genes by gene identifier, COG number, organism, and function to reduce confusion caused by the common practice of assigning different gene names to homologous genes that have related functions. To evaluate the conservation and diversity of C1 oxidation pathways in SAR11 we examined the distribution of genes for C1 metabolism among three SAR11 genomes of the Group Ia subclade, and these genes were found in all three SAR11 Ia genomes (Table 1). A survey of the global ocean survey (GOS) metagenomic data for SAR11 genes involved in C1 metabolism also supported the conclusion that these genes occur frequently in SAR11 genomes. Averaged across ocean sampling locations worldwide, the ratio of SAR11 C1 genes, relative to SAR11 *recA*, ranged from 0.2 to 2.1 (Fig. S1 and Table S2).

**Table 1:** Distribution of genes involved in C1 metabolism among three SAR11 Ia genomes.

Genes for C1 oxidation and methylovory		HTCC 1062	HTCC1002	HTCC7211
THF-linked oxidation	formate dehydrogenase, alpha subunit (*fdhF*)	+	+	+
	NAD-dependent formate dehydrogenase, beta subunit (*fdsB*)	+	+	+
	formate dehydrogenase, chain D (*fdhD*)	+	+	+
	molybdopterin-guanine dinucleotide biosynthesis protein A (*mobA*)	+	+	+
	molybdopterin biosynthesis protein (*moeA*)	+	+	+
	formate-THF ligase (*fhs*)	+	+	+
	methylene-THF reductase (*metF*)	+	+	+
	bifunctional methylene-THF dehydrogenase-methenyl-THF cyclohydrolase (*folD*)	+	+	+
methanol oxidation	iron-containing alcohol dehydrogenase (Fe-ADH)	+	+	+
methylamine oxidation	glutamine synthetase III (*glnT*)	+	+	+
	putative N-methylglutamate synthase (*glxBCD*)	+	+	+
	putative N-methylglutamate dehydrogenase (*soxBDAG)*	+	+	+
GBT oxidation	betaine-homocysteine methyltransferase (*bhmT*)	+	+	+
	sarcosine dehydrogenase (*sardh*)	+	+	+
	dimethylglycine dehydrogenase (*dmgdh*)	+	+	+
AMTs	glycine system cleavage T-protein (*gcvT*)	+	+	+
	dimethylsulfoniopropionate-dependent demethylase (*dmdA*)	+	+	+
	putative aminomethyltransferase	+	+	+
GSH dependent pathway	glutathione-dependent formaldehyde activating enzyme (*gfa*)	-	-	+
	glutathione-dependent formaldehyde dehydrogenase (GD-FALDH)	-	-	+
	S-formyl-glutathione hydrolase (FGH)	-	-	+

The phylogenomics pipeline HAL and manual searches were used to detect orthologs among the genomes. Genes for C1 oxidation were present in all three genomes. HTCC7211 possesses three genes for the glutathione (GSH) dependent C1 oxidation pathway that are not present in the other two SAR11 Ia genomes.

Central to the process of C1 and methylated compound oxidation in SAR11 Ia is the tetrahydrofolate (THF)-linked oxidation pathway, which oxidizes C1 units to CO_2_, yielding energy in the form of reduced nucleotides and ATP (Fig. 2). The gene products of *metF* (SAR11_1264), *folD* (SAR11_0307) and *fhs* (SAR11_1285) are predicted to catalyze early steps in the methyl-THF linked oxidation pathway (Fig. 2A). The genes *fdhF* (SAR11_0679), *fdsB* (SAR11_0680) and *fdhD* (SAR11_0681; Fig. 1A) are predicted to encode subunits of formate dehydrogenase (FDH), which catalyzes the final step in the pathway, the oxidation of formate to CO_2_ [30], [31] (Fig. 2A). Gene *mobA* (SAR11_0684) and *moeA* (SAR11_0685) are predicted to encode proteins for the synthesis of a molybdenum cofactor, which is required for the activity of most bacterial molybdoenzymes, such as FDH [32]-[34].

Putative genes that encode C1 oxidation activities are found in many bacterial phyla, archaea and eukaryotes. We used the phylogenomic pipeline HAL [35] to study the distribution of C1 oxidation genes in the class *Alphaproteobacteria* and found evidence for many of these genes throughout the genomes investigated (Fig. S2). For example, homologs of *fdhF* and *fdhD* were observed in 77 and 58 of 127 genomes, respectively. However, outside of the methylotrophs, which can grow using C1 compounds as a sole carbon and energy source, C1 oxidation has received relatively little attention. FDH genes have been reported in *Rhizobium japonicum* and *Agrobacterium tumefaciens* [36]-[38]. Interestingly, several non-methylotrophs, including *A. tumefaciens*, are capable of utilizing methylamine as a nitrogen source, but not as a sole carbon source. As with SAR11 Ia, these bacteria contain the proposed THF-linked C1 oxidation pathway and lack specialized pathways for the assimilation of C1 compounds [8], [36]. These observations suggest that, as we report below for SAR11 Ia strains, *R. japonicum* and *A. tumefaciens*, and probably many other bacteria, may utilize C1 and methylated compounds for energy production.

**Figure 3:**
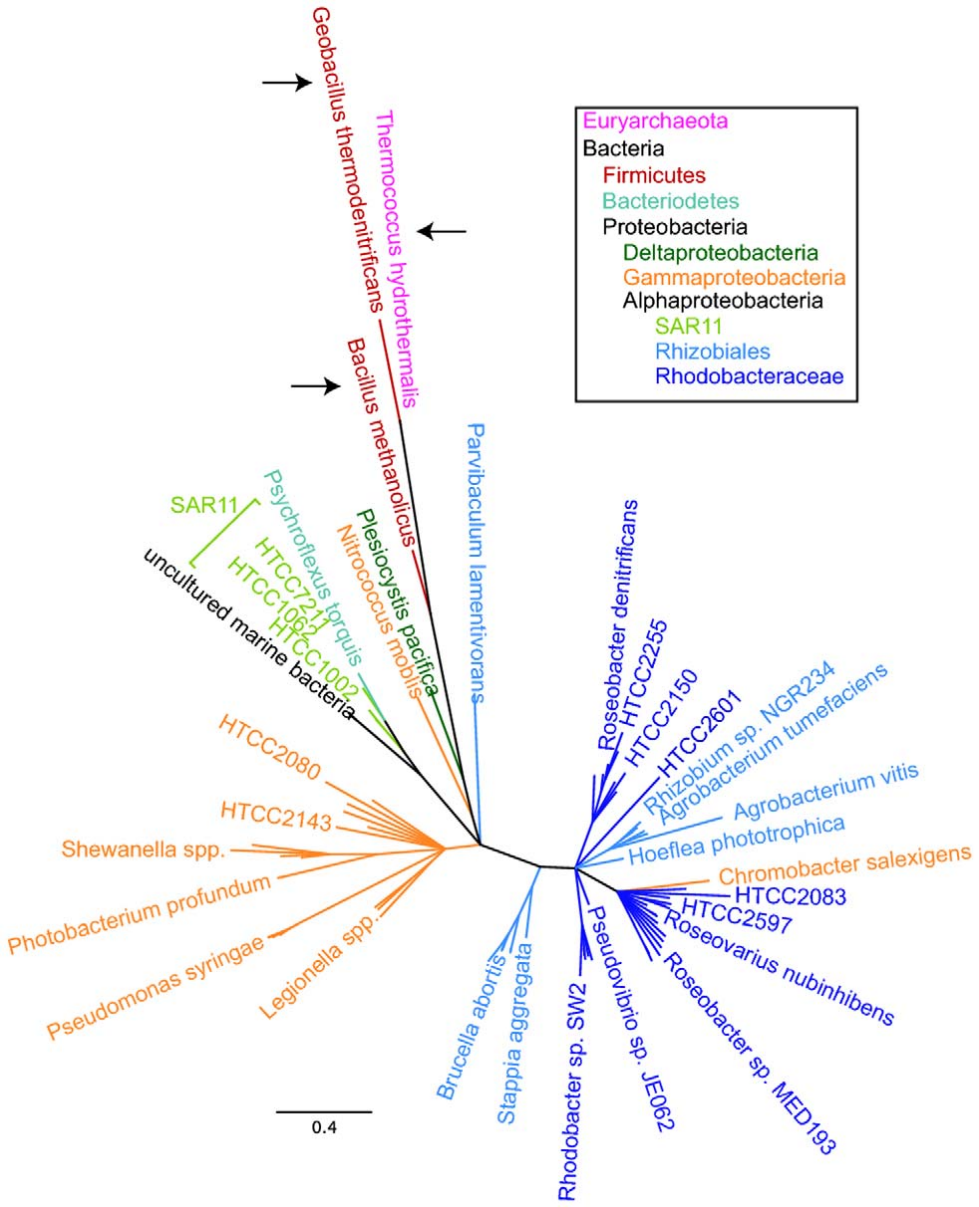
Phylogenetic tree of Fe-ADH proteins. Coloration is according to 16S rRNA gene phylogeny, as shown in the boxed legend. Bootstrap values were omitted for clarity; nodes with less than 60% support were collapsed. Arrows indicate Fe-ADH proteins for which methanol dehydrogenase activity has been demonstrated experimentally. Scale bar  = 0.4 changes per position.

All three SAR11 Ia genomes possess an alcohol dehydrogenase (ADH) gene (SAR11_1287; Fig. 1B) that was initially annotated as methanol dehydrogenase. We propose that this enzyme catalyzes the oxidation of short chain alcohols, including methanol, to the corresponding aldehydes (Fig. 2B). In the case of methanol, the oxidation product is formaldehyde, which can be converted to CO_2_ by the methyl-THF linked oxidation pathway described above. This gene encodes a member of the iron-containing alcohol dehydrogenase (Fe-ADH, PF00465) protein family found in many microorganisms. Oxidation of methanol rarely is observed in non-methylotrophic bacteria that lack the classical pyrroloquinoline-quinone (PQQ)-containing methanol dehydrogenase. Methanol dehydrogenase activity has been confirmed in ADH proteins from some *Firmicutes* and Archaea [39]-[41]. A phylogenetic tree of the Fe-ADH proteins from three SAR11 Ia strains (Fig. 3, light green) and closely related homologs from other organisms shows that the proteins with demonstrated methanol dehydrogenase activity (arrows) are more highly diverged than their close relatives, and that the SAR11 Fe-ADH proteins branch nearby (Fig. 3). As we report below (Tables 2 and 3), physiological evidence from ATP assays supports the conclusion that the SAR11 Fe-ADH is not specific for methanol oxidation, but can oxidize other short chain primary alcohols as well. Similar conclusions were reported previously for other bacterial ADH genes in the same family [42]-[44]. Only short-chained, primary alcohols (methanol, ethanol, and 1-propanol) stimulated ATP production in HTCC1062.

**Table 2:** ATP response of starved cells to addition of various alcohols^a^.

Test compounds^b^	samples	Cellular ATP content (Mean ± SD; zeptogram cell^-1^)
* ethanol	T	57±8
	N	14±3
	P	135±15
* 1-propanol	T	47±6
	N	19±4
	P	41±3
2-propanol	T	10±1
	N	11±2
	P	26±1
1-butanol	T	25±7
	N	23±9
	P	35±7
2-pentanol	T	13±2
	N	11±2
	P	25±9
iso-amyl alcohol	T	18±5
	N	14±2
	P	40±8

a. For each assay, cells were grown in media containing the test compound, then washed and starved for 20 hrs. Cellular ATP content was measured after cells were exposed for 2 hrs to the test compound (T), to no compound added (N), and to pyruvate (P; positive control to confirm metabolic activity of cells).

b. Asterisk indicates statistical significance (p-value <0.01) between “no compound added” and “test compound” treatments.

**Table 3:** ATP response of starved cells to addition of C1 and methylated compounds^a^.

Test compounds^b^	samples	Cellular ATP content (Mean ± SD; zeptogram cell^−1^)
formate	T	32±3
	N	29±8
	P	221±4
* methanol	T	48±0
	N	16±3
	P	160±8
* formaldehyde	T	33±6
	N	14±1
	P	77±5
* DMSP	T	23±3
	N	16±1
	P	163±7
* methylamine	T	27±1
	N	18±0
	P	145±10
* glycine betaine	T	41±1
	N	23±3
	P	132±3
* TMAO	T	63±5
	N	26±2
	P	148±10

a. For each assay, cells were grown in media containing the test compound, then washed and starved for 20 hrs. Cellular ATP content was measured after cells were exposed for 2 hrs to the test compound (T), to no compound added (N), and to pyruvate (P; positive control to confirm metabolic activity of cells).

b. Asterisk indicates statistical significance (p-value <0.01) between “no compound added” and “test compound” treatments.

A predicted GBT degradation pathway operon was identified in the SAR11 Ia genomes. In this pathway, the three N-methyl groups of GBT are removed in sequence by three enzymes: betaine-homocysteine methyltransferase (BHMT), dimethylglycine dehydrogenase, and sarcosine dehydrogenase (encoded separately by SAR11_1173, SAR11_1253, and SAR11_1221 in HTCC1062). These methyl groups have different predicted fates. The first methyl group is transferred to methionine by BHMT, an enzyme that is common and well studied in mammals, but is rarely found in prokaryotes [45], [46]. The second and third methyl groups are predicted to be transferred to THF in reactions that are coupled to partial oxidation and then further oxidized to CO_2_ by the THF-linked oxidation pathway described above (Fig. 2A, C). A previous study showed that the uptake and metabolism of GBT by bacteria-sized organisms in seawater was correlated with salinity [47]. Consistent with that study, metagenomic analysis showed relatively constant ratios of SAR11 GBT gene frequencies in ocean populations, and absence of these genes in metagenomes from fresh water (Table S2, ID GS20).

Genes for methylamine dehydrogenase are missing from SAR11 Ia genomes, but they contain genes necessary to encode an alternative, glutamate-mediated pathway for methylamine oxidation [48]. Latypova and colleagues showed that N-methylglutamate synthase (NMGS) and N-methylglutamate dehydrogenase (NMGD), encoded by *mgsABC* and *mgdABCD* in *Methyloversatilis universalis*, participate in methylamine oxidation [48]. When the *M. universalis* NMGS protein sequences were blasted against the predicted HTCC1062 proteome, it returned a *glxBCD* operon (SAR11_1313-1315) as the top hit. This operon is annotated as a glutamate synthase and shares gene order with the *M. universalis* NMGS operon. We hypothesize that this operon may encode a holoenzyme that has NMGS activity. NMGS catalyzes transfer of a methyl group from methylamine to glutamate to produce N-methyglutamate as a product.

We postulate that the next step in the pathway, oxidation of N-methyglutamate to glutamate and 5, 10-methylene-THF, with the reduction of FAD to FADH_2_, is catalyzed by the products of SAR11 genes that are annotated as heterotetrameric forms of sarcosine oxidase (Fig. 2D). Two paralogous operons of four sarcosine oxidase genes, *soxBDAG* (SAR11_1064-1067) and *soxB_2_D_2_A_2_G_2_* (SAR11_1281-1284), are located in separate locations of HTCC1062 genomes with the same gene orders and conserved functional domains as those encoding NMGD in *M. universalis* (Fig. 1C). Metagenome surveys showed that these two sets of paralogs are highly represented in SAR11 populations globally (Fig. S1). We postulate that one of these sets of paralogs encodes the sarcosine oxidase holoenzyme, and the other encodes NMGD; however, which operon encodes which function is unclear. Heterotetrameric sarcosine oxidases (a.k.a sarcosine dehydrogenase) participate in the GBT degradation pathway by converting sarcosine to glycine. In HTCC1062, SAR11_1304 is predicted to encode the monomeric form of sarcosine oxidase, and is adjacent to two genes annotated as an aminomethyltransferase (AMT) and a GBT transporter (SAR11_1303 and SAR11_1302; Fig. 1E). The presence of two paralogous sarcosine oxidase operons and an individual gene encoding a monomeric sarcosine oxidase is noteworthy considering that paralogs are rare in highly compact SAR11 genomes [28]. We postulate that SAR11 Ia strains may use these genes for the reactions described above and possibly for the demethylation of unknown substrates in oxidation reactions that are likely to produce the intermediate 5, 10-methylene-THF [49].

A different pathway for methylamine demethylation that involves the intermediate gamma-glutamylmethylamide was also postulated by Latypova and colleagues [48]. We found the homolog of gamma-glutamylmethylamide synthetase (GMAS) from this pathway in HTCC1062, which was annotated as *glnT*, a glutamine synthetase III (SAR11_1316, Fig. 1C) and shares the conserved Gln-synt_C domain (Pfam PF00120.17) with GMAS. However, the complete pathway has not been identified and the enzyme was considered to be not essential for oxidation of methylamine in *M. universalis* [48].

**Figure 4:**
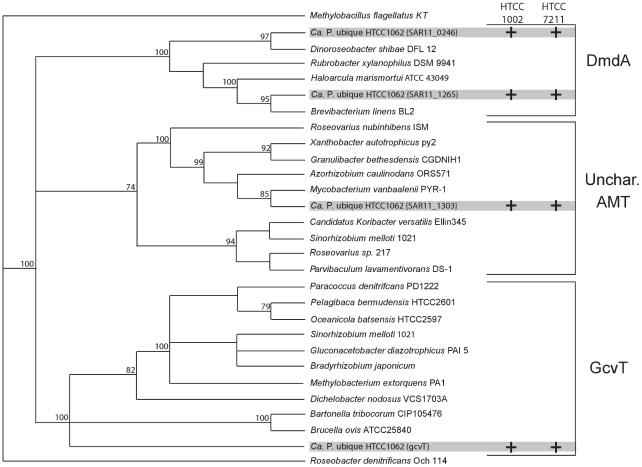
Phylogeny of SAR11 AMT proteins. Four paralogous AMTs in HTCC1062 were placed into three functional subgroups: DmdA-like, GcvT, and an AMT of unknown function. All four AMTs were also identified in HTCC1002 and HTCC7211 genomes. This phylogenetic tree was generated using the neighbor-joining method. Bootstrap values are based on 100 iterations.

The three SAR11 Ia genomes include a family of paralogous AMT genes (Fig. 1E). Phylogenetic analysis provided strong support for placing the four AMT genes from HTCC1062 into three functionally distinct subgroups (Fig. 4). SAR11_0666 encodes the glycine cleavage system T-protein (GcvT), part of the glycine cleavage multi-enzyme complex (GCV), which is present in most bacteria and mitochondria [50]. GCV catalyzes the degradation of glycine to form 5, 10-methylene-THF, CO_2_ and NH_3_ [51] (Fig. 2F). SAR11_0246 encodes DmdA, a member of the AMT protein family that catalyzes removal of the first methyl group from DMSP to produce methylmercaptopropionate (MMPA) in a pathway for DMSP catabolism [52], [53] (Fig. 2G). However, the enzyme that subsequently demethylates MMPA to mercaptopropionate (MPA) has not been identified.

SAR11_1265 and SAR11_1303 are annotated as probable AMTs with unknown substrates. Three genes (*opuAB, opuAA,* and *opuAC*) are located near SAR11_1303 and encode putative homologs of the GBT transport system permease protein, GBT transport ATP-binding protein and substrate-binding region of ABC-type GBT transport system (Fig. 1E). Therefore, we postulate that SAR11_1303 may encode an AMT involved in GBT metabolism. Notably, SAR11_1265 is adjacent to the putative *metF* (SAR11_1264), encoding a homolog of methylene-THF reductase. MetF in *Methylobacterium chloromethanicum* CM4 enables the oxidation of methyl-THF to methylene-THF [20]. SAR11_1265 is closely related to SAR11_0246 (*dmdA*) (Fig. 4), and was observed at similar abundances in GOS metagenomic analysis (Fig. S1), leading us to speculate that this gene might catalyze removal of the second methyl group in the DMSP degradation pathway. All of the four AMTs tended towards a 1∶1 ratio with single-copy gene *recA* (Fig. S1), suggesting that these genes may be part of the SAR11 Ia core genome.

Another gene, SAR11_0621, was initially annotated as an AMT, but later was identified as *ygfZ*, which encodes a folate-binding protein. Studies on YgfZ in *Escherichia coli* and *Arthrobacter globiformis* revealed THF-binding folds in the structure of this protein, but otherwise little similarity to the GcvT family of AMT proteins [54], [55]. Recent studies indicated that YgfZ in *E. coli* has methylase activity [56] or may be involved in the regulation of C1 metabolism [54]. There is currently no direct evidence for a role in C1 metabolism for YgfZ in SAR11.

Some of the three SAR11 Ia genomes had additional genes implicated in C1 metabolism (Table 1). Genes encoding glutathione-dependent formaldehyde activating enzyme (GFA), glutathione-dependent formaldehyde dehydrogenase (GD-FALDH) and S-formyl-glutathione hydrolase (FGH) are present in HTCC7211, but not in HTCC1062 or HTCC1002. In *Paracoccus denitrificans*, these proteins catalyze the well-studied glutathione (GSH) dependent pathway that converts formaldehyde to CO_2_ [7], [57]-[59]. We note that some of the predicted C1 oxidation functions of these genes are redundant with genes for the THF-linked oxidation pathway that are also present in the HTCC7211 genome; however, unlike strain HTCC1062, strain HTCC7211, an isolate from the oligotrophic Sargasso Sea, is predicted to be able to oxidize formaldehyde by using GFA to catalyze the first step in the pathway.

Methane monooxygenases, which catalyze the first reaction of methane oxidation pathways, were not present in any SAR11 genome, indicating that these cells are unlikely to be methane oxidizers. Karl and coworkers reported that methane is produced from methylphosphonate in seawater by the activity of the microbial C-P lyase pathway [60]. This pathway is present in the genome of HTCC7211, but there are no known metabolic pathways by which methyl groups from methylphosphonate can be diverted into THF-linked C1 oxidation.

### Direct Experimental Evidence for C1 Oxidation in SAR11 Strain HTCC1062

Genome analysis suggested that C1 metabolism evolved in SAR11 Ia for energy production, rather than as a means to accumulate biomass. Testing this hypothesis was challenging because SAR11 Ia cells require a variety of unusual organic growth factors and cannot be cultured on “sole” carbon sources, a common paradigm in microbiology. Some required compounds, notably glycine, can be oxidized to produce energy as well as being needed for biomass production [61]. Thus, we were not surprised when the addition of various C1 and methylated compounds did not change growth rates or yields of cultures (data not shown). To test for C1 oxidation activities predicted by genome analysis, we turned to direct measurements of energy production and substrate oxidation.

Measurements of cellular ATP content supported the conclusion that HTCC1062 can produce energy from a wide variety of C1 and methylated compounds (Table 3). ATP levels were assayed in HTCC1062 cells that were first grown in the presence of C1 and methylated compounds to be certain that pathways involving C1 oxidation genes were induced. Concentrations of compounds used for ATP assays and ^14^C experiments (below) were determined in advance by growth assays, as described in the materials and methods. Pyruvate, which was shown previously to be actively metabolized as an energy and carbon source by HTCC1062 cells [62], was used as a positive control. ATP content increased in cells incubated with all of the methylated compounds tested with the exception of formate, relative to negative controls. Methanol and TMAO caused the greatest increases in ATP content (3- and 2.4-fold, respectively). We speculate that formate did not enhance ATP levels because it was not transported into cells.

**Figure 5:**
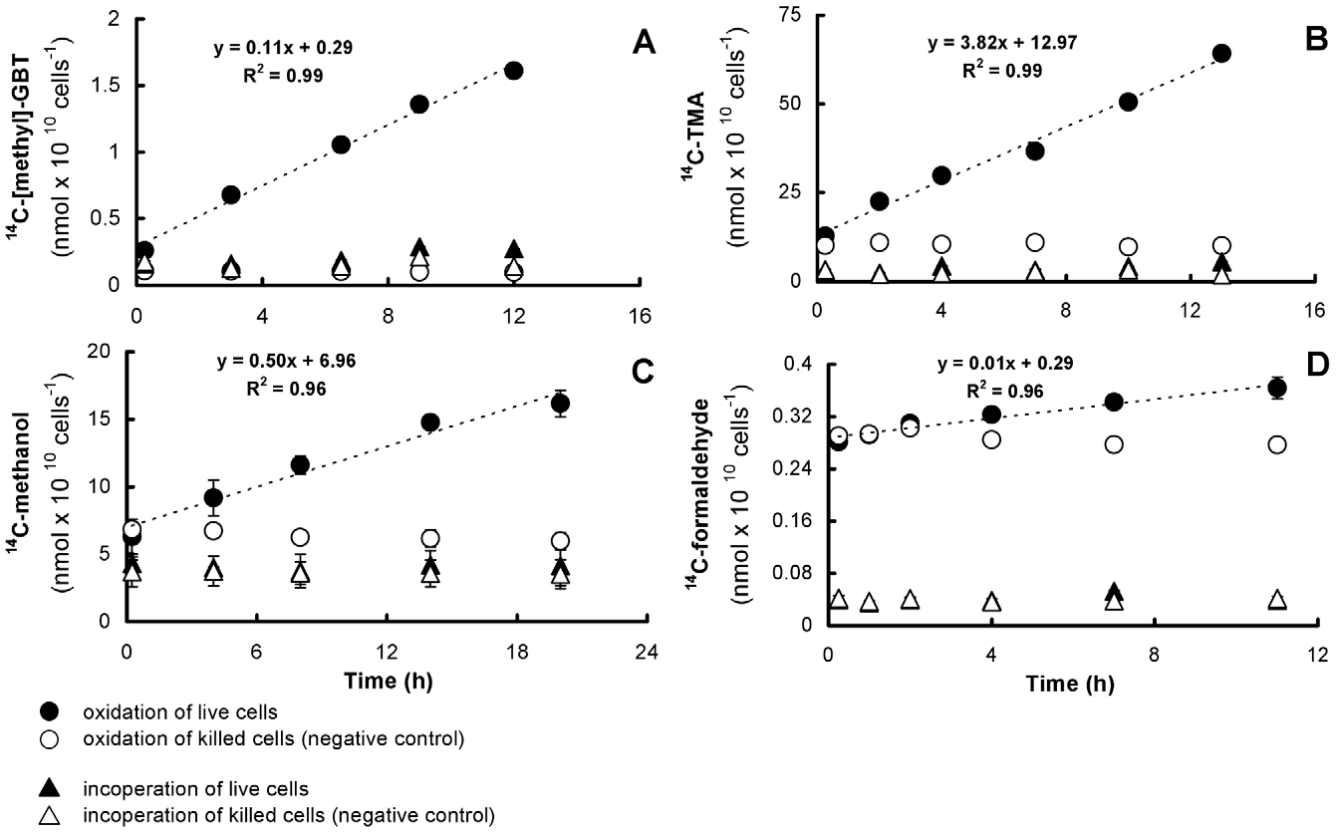
^14^C-labeled compound utilization by HTCC1062 in culture. HTCC1062 Cells from log phase were collected and resuspended in artificial seawater media (ASW). Radioisotope assays were conducted at room temperature (22°C) in ASW amended with (A) 1 µM ^14^C-[methyl]-GBT; (B) 5 µM ^14^C-TMA; (C) 20 µM ^14^C-methanol; or (D) 100 nM ^14^C-formaldehyde. Where not visible, error bars are smaller than the size of the symbols.

Consistent with predictions from the genome sequence, radioisotope studies with ^14^C-[methyl]-GBT, ^14^C-TMA, ^14^C-methanol and ^14^C-formaldehyde demonstrated that HTCC1062 oxidized methyl groups from methylated compounds and C1 compounds to CO_2_, but incorporation of the compounds into biomass was negligible (Fig. 5). Among these compounds, the oxidation rate was fastest with 5 µM ^14^C-TMA (3.82 nmol×10^10^cells^−1^×h^−1^), while 100 nM ^14^C-formaldehyde was oxidized to ^14^CO_2_ at the lowest rate (0.01 nmol×10^10^ cells^−1^×h^−1^), likely reflecting the lack of formaldehyde activating enzyme in HTCC1062. The rate of 20 µM methanol oxidized to ^14^CO_2_ was 0.50 nmol×10^10^ cells^−1^×h^−1^.

**Figure 6:**
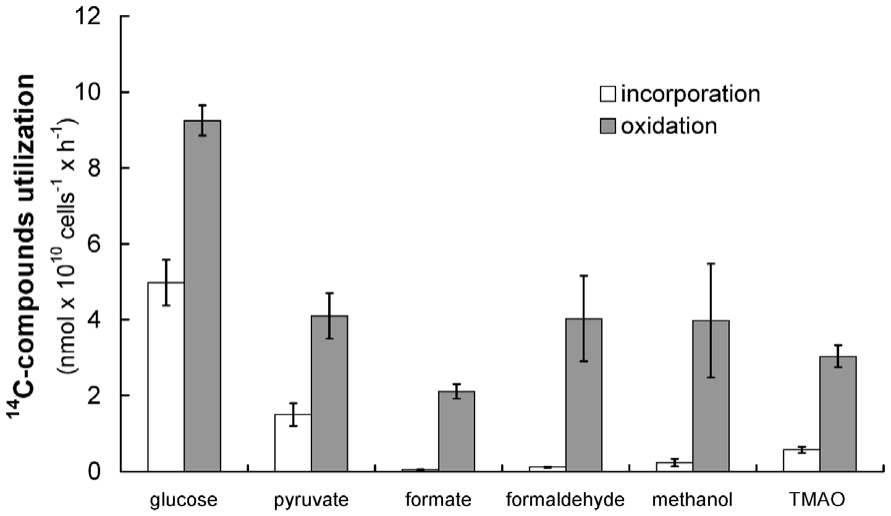
Utilization of ^14^C-labeled C1 and methylated compounds by bacterioplankton in the western Sargasso Sea. The oxidation and incorporation rates were calculated from the initial linear part of each curve. Rate of ^14^C-compound oxidation to ^14^CO_2_ (▪); rate of ^14^C-compounds incorporation into biomass (□).

The ability of cultured *Ca.* P. ubique to oxidize C1 compounds, and the conservation of genes for C1 metabolism in streamlined SAR11 genomes, suggests that C1 oxidation pathways may contribute significantly to the energy budget of these cells in nature. To examine this hypothesis, we measured the potential rates at which ^14^C-labeled C1 and methylated compounds were oxidized and incorporated into biomass by cells concentrated from the Sargasso Sea upper euphotic zone during the period of summer stratification. Long-term time series measurements have shown that SAR11 cells range from 30-40% of cells at this ocean site, and that the distinctive microbial community that forms in the upper euphotic zone during the summer is dominated by the SAR11 Ia subclade [25], [29]. The data from bacterioplankton populations collected in the western Sargasso Sea revealed rates of carbon assimilation and oxidation of the same order observed with pure SAR11 cultures in the laboratory (Fig. 6 and [62]). Similar methanol oxidation rates (∼0.8 – 2.5 nmol×10^10^ cells^−1^×h^−1^) were measured in off-shelf northeast Atlantic seawaters [14], which are also dominated by SAR11 cells [25]. Notably, in the natural bacterioplankton community less than 6 % of the ^14^C-methanol, 2 % of ^14^C-formate, and 3 % of ^14^C-formaldehyde were assimilated into biomass, with the remainder oxidized to CO_2_ by the natural community. In contrast, 35 % of glucose and 27 % of pyruvate were assimilated into bacterioplankton biomass in the field experiments (Fig. 6), similar to the fraction of organic carbon assimilation into biomass observed previously with growing SAR11 cultures and typical of bacterial organic carbon assimilation in general [62]. One noteworthy difference between the results obtained with cultured strain HTCC1062 and measurements made in the field is the higher rate of formaldehyde oxidation in the field study. During comparative genome sequence analysis the protein GFA (formaldehyde activating enzyme) was noted in the HTCC7211 genome (Table 1), an isolate obtained from the Sargasso Sea. It may be that ecotypes of SAR11 found in the Sargasso Sea summer upper euphotic zone microbial community have a potential to oxidize formaldehyde that is not found in coastal isolates. Overall, these findings suggest that energy production by C1 oxidation, rather than methylotrophy, in which a large fraction (i.e., 62 %; [63]) of the C1 compound is incorporated into biomass, is the predominant mode of C1 oxidation in surface waters of temperate oligotrophic oceans.

### Conclusions

It is a paradox that the SAR11 clade evolved small genomes while becoming the most successful heterotrophs known, because DOC is a complex substrate that would appear to require complex metabolic pathways for oxidation. The most obvious solution to this conundrum is substrate specialization by SAR11 and other heterotrophic species in microbial communities, dividing the oxidative side of the carbon cycle into niches. In this report we show that a considerable part of the SAR11 Ia genomes is devoted to C1 metabolism that produces energy but not biomass. C1 and methylated compounds have not generally been regarded as a large fraction of the marine carbon cycle, but there have been a number of reports of C1 compounds and C1 oxidation activity in marine systems [14], [47]. We speculate that C1 metabolism evolved in SAR11 because it gave these cells the ability to catabolize a variety of compounds – ranging from low molecular weight photolysis products to osmolytes carrying multiple methyl groups, such as DMSP and GBT. DMSP catabolism in SAR11, and its use as a source of reduced sulfur for growth, has been the subjects of previous studies that did not examine the fate of methyl groups [53], [64].

The concept of cells producing energy but not biomass from C1 compounds has received little attention. The best analogy is the term “carboxidovory”, which was coined to describe the metabolism of cells that produce energy by oxidizing carbon monoxide, and to distinguish them from “carboxidotrophs” [65], [66], which can use carbon atoms from carbon monoxide for the net assimilation of biomass. Based on this precedent, we propose a new term, “methylovores”, to distinguish cells, such as SAR11 and likely many other bacteria, that can utilize C1 compounds as a source of energy, from methylotrophs, which are able to use C1 compounds as sole sources of energy and carbon.

Phylogenomic studies have placed the mitochondria deep within the *Alphaproteobacteria*, close to *Rickettsiales* and the SAR11 clade [67]. Although respiration is a central feature of mitochondria that is assumed to have driven the original endosymbiotic event, mitochondria make complex contributions to cellular metabolism, including vitamin biosynthesis [68], and the metabolism of C1 compounds [69], [70], which evidence supports as being present in the protomitochondrion [71]. In light of the data presented here it is not unreasonable to speculate that the C1 metabolism of mitochondria was contributed by free-living methylovorous ancestors of modern SAR11.

The findings we report show that SAR11 Group Ia strains can produce cellular energy from a broad range of C1 and methylated compounds by oxidative metabolism that we here refer to as methylovory to distinguish it from methylotrophy. The data we present also show that the potential for methylovory was highly expressed in a natural oligotrophic ocean surface microbial community dominated by SAR11. Based on these findings, we speculate that C1 oxidation pathways contribute significantly to the marine carbon cycle and in part explain the broad success of SAR11.

## Materials and Methods

### Bioinformatics analysis and phylogeny

Gene calls and functional assignment of SAR11 Group Ia genomes were performed by an automated pipeline at Oregon State University's Center for Genome Research and Biocomputing (http://bioinfo.cgrb.oregonstate.edu/microbes/index.html). Conserved domains were determined with Pfam [72]. KEGG [73] and MicrobesOnline [74] were used for predicting physiological and metabolic pathways.

For the phylogeny of the Fe-ADHs, Pfam was used to confirm the orthology of the Fe-ADH domain in the three SAR11 strains with that of the known methanol dehydrogenase genes (Pfam Fe-ADH PF00465). The phylogenetic tree was then constructed using these six sequences and those of over 70 of the 100 top hits identified using PSI-BLAST with the HTCC1062 gene (YP_266695) as the query sequence. Sequences were aligned using MUSCLE [75], [76], and manually edited to remove columns with >85% gaps. Substitution modeling was completed using ProtTest [77]. The alignment was then analyzed with RAxML [78] using the WAG substitution model and nodal support was estimated based on 100 bootstrap replications.

Phylogeny of the AMTs was performed by MicobesOnline website and MacVector software. The top 10 matched protein sequences of each AMT (YP_265671, YP_266089, YP_266673, YP_266710) were downloaded using BLAST tools from MicrobesOnline. AMTs were aligned using the ClustalW module of MacVector version 10.5.2, and Phylogenetic trees were constructed using the neighbor-joining method. One-hundred bootstrap replicates were used to estimate the robustness of branches in the phylogenetic tree. Accession numbers are in Table S3.

Homology assessment of methylovory genes was completed using the HAL pipeline [36], [79] (http://aftol.org/pages/Halweb3.htm; http://sourceforge.net/projects/bio-hal/) followed by manual examination of predicted orthologous clusters. For this study, the HAL pipeline directed the following analyses: Protein sequences from 127 publically available *Alphaproteobacteria* genomes (for a complete list, see [67]) were imported in FASTA format from IMG (http://img.jgi.doe.gov) and subjected to all vs. all BLASTP with the output E-values provided to the program MCL [80], which grouped proteins into orthologous clusters using 13 inflation parameters from 1.1 – 5.0. For homology assessment in Figure S2, we examined clusters formed at the relatively conservative 3.0 inflation parameter, and thus these numbers can be considered as lower estimates of total homologs.

### Metagenomic analysis

The reciprocal best BLAST (RBB) approach [81] was used to identify environmental fragments in the global ocean survey (GOS) database originating from SAR11 and encoding genes related to C1 and methyl group oxidation. The amino acid sequences for the genes discussed in this paper were searched with BLASTP (e-value threshold  = 1×10^−5^) against peptide sequences called from GOS metagenomic nucleotide reads. The taxonomies of matching peptides were determined by querying the peptide against the NCBI non-redundant protein database (NR) using BLASTP (e-value threshold  = 10). Sequences were only retained if this second search against NR found one of the three *Ca.* P. ubique strains to be the best match to the environmental peptide. Peptides were lastly searched with BLASTP (e-value threshold  = 1×10^−5^) against all *Ca.* P. ubique strain HTCC1062 amino acid sequences to ensure that the initial *Ca.* P. ubique query protein, rather than a paralog, was the best match to the environmental peptide sequence. The number of environmental peptides passing the RBB test, or hits, per gene at each GOS sampling site were normalized to the length of the single-copy essential gene *recA* and the number of hits to *recA* at the same sampling site using the formula P*_g_* = (L*_recA_* / L*_g_*)×(H*_g_* / H*_recA_*), where P*_g_* is the percentage abundance related to *recA* for gene *g*, L*_recA_* is the length of *recA* in base-pairs (bp), L*_g_* is the length of gene *g* in bp, H_g_ is the number of hits for gene *g*, and H*_recA_* is the number of hits to *recA* [82]. GOS sampling sites that had fewer than five hits to *recA* were removed from downstream analysis.

### ATP measurements

HTCC1062 cultures for ATP assays were grown in autoclaved, filtered seawater amended with 1 mM NH_4_Cl, 100 µM KH_2_PO_4_, 1 µM FeCl_3_, 80 µM pyruvate, 40 µM oxaloacetate, 40 µM taurine, 1 µM GBT, 50 µM glycine, 50 µM methionine, and excess vitamins [62]. C1 and methylated compounds to be tested were added as follows: 1 µM GBT, 20 µM formate, 10 µM methylamine, 5 µM TMAO, 1 µM DMSP, 20 µM methanol and 100 nM formaldehyde. The concentration of GBT used in the assay was the same as routinely used in *Ca.* P. ubique growth media (1 µM). The concentrations of other compounds utilized in assays were chosen by testing the growth of HTCC1062 with differing concentrations of each compound and selecting concentrations that were not inhibitory (Fig. S3). Cultures were grown in the dark at 16°C. When cultures entered early stationary phase (∼3×10^8^ cells mL^−1^), 10 mL of each culture were collected and cells were harvested via centrifugation (50 min at 43,700 *g*, 10°C). Following centrifugation, cells were washed twice in artificial seawater (ASW) [62] and finally resuspended in 5 mL ASW. Then cells were distributed into 1.7 mL tubes (500 µL in each tube) and starved overnight (20 hours in the dark, 16°C). Test treatments and controls were done in triplicate with identical cell suspensions. After 2 hours incubation in the dark, ATP content was measured using a luciferase-based assay (BactTiter Glo, Promega, Madison, WI) as follows: 90 µL of BactTiterGlo reagent were dispensed into white 96 well plates (Tissue culture-treated, BD Biosciences, San Jose, CA). 20 µL of each sample were added per well, and luminescence was measured after 4 min using a multi-functional plate reader (TECAN, Infinite M200) with a 1 s integration and 10 ms settle time. An ATP standard curve was used to calculate the concentration of ATP in the samples. Student's t-test was used to assess statistical significance (p-value<0.01) between controls and treatments. The ATP measurements for other alcohols (20 µM ethanol, 20 µM 1-propanol, 20 µM 2-propanol, 20 µM 1-butanol, 20 µM 2-pentanol, and 20 µM iso-amyl alcohol) were performed as described above.

### Radioisotope assays

To distinguish between ^14^C-labeled compounds incorporated into biomass and oxidized to ^14^CO_2_, a method was devised for volatile ^14^C compounds (e.g., ^14^C-labeled methanol). Trichloroacetic acid (TCA)-precipitation was used to assess ^14^C-incorporation into cellular material, and ^14^CO_2_ was precipitated by addition of NaOH, Na_2_CO_3_ and BaCl_2_, forming Ba^14^CO_3_ and BaOH. Filtration removed unincorporated ^14^C compounds and BaOH, thus minimizing potential quenching effects of the base.

For experiments with radioisotopes, HTCC1062 was grown in seawater medium as described above for ATP measurements. Cultures were amended with unlabeled test compounds to induce activities (5 μM TMA, 20 μM methanol, or 100 nM formaldehyde; 1 μM GBT was in the seawater medium). Cells were harvested in log phase by centrifugation (1 hour at 43,700 *g*, 10°C) and resuspended in ASW to about 4×10^7^ cells mL^−1^. Negative controls (“Killed”) were incubated in 10% formalin for 1 hour before the addition of the isotope to the sample. 1 µM ^14^C-[methyl]-GBT, 5 µM ^14^C-TMA, 20 µM ^14^C-methanol, or 100 nM ^14^C-formaldehyde were added to both “Live” and “Killed” culture samples. Inoculated cultures (4 mL) were aliquoted into 40 mL sealed vials. Samples were incubated at room temperature. At each time point, reagents were added to cultures using syringes inserted through stoppers. For ^14^C-incorporation, 2.2 mL 100% w/v cold TCA was added; for trapping ^14^CO_2_, 1 mL 1N NaOH, 0.5 mL 0.1 M Na_2_CO_3_ and 1 mL 1 M BaCl_2_ were added. All samples were collected by filtration after incubation at 4°C for 12 hrs. Filters were transferred to vials containing 15 mL Ultima Gold™ XR scintillation fluid (Perkin-Elmer) and kept in dark overnight before counting (Beckman LS-6500 liquid scintillation counter).

For field studies of radiolabeled compound utilization by bacterioplankton in the western Sargasso Sea, seawater was collected from 10 m at Bermuda Hydrostation S using Niskin bottles, and transferred to an acid-washed, autoclaved polycarbonate carboy. For each experiment, microbial plankton were concentrated from 80 L seawater by tangential flow filtration to a final volume of 600 mL, and isotopic labeling was carried out as described above. The concentrations of the tested compounds were 3 µM glucose, 1.7 µM pyruvate, 0.3 µM formate, 1.1 µM formaldehyde, 50 µM methanol and 0.5 µM TMAO. A high concentration of methanol was used because the specific activity of the labeled compound was very low. Some unexpected evaporation of the radio-labeled compounds occurred during transport causing some variability between concentrations of the other compounds used. Nevertheless, in all cases the concentrations used were likely to be substantially higher than typical seawater concentrations, and the results represent potential rates of compound utilization.

## Supporting Information

Figure S1 **The abundance of SAR11 C1 metabolism genes in GOS data, relative to SAR11** ***recA*** **genes.** Genes were identified as SAR11 by a reciprocal best BLAST (RBB) approach. SAR11 C1 genes with frequencies less than SAR11 *recA* (<1x) may indicate that only subpopulations of SAR11 cells possess that gene; genes greater than 1x suggest that multiple copies of that gene are present per cell. Boxes encompass points between the 25th and 75th percentiles, with the median represented as a thick horizontal line. Whiskers span the minimal distance needed to include all points within 1.5 x the interquartile range beyond the interquartile boundary, with points outside of this range rendered individually as circles. For each gene, n = 40. Abbreviations: adj., genomically adjacent genes.
(TIF)

Figure S2 **Distribution of C1 gene homologs throughout the** ***Alphaproteobacteria*** **.** The number of genomes containing homologs of C1 oxidation genes reported by gene and divided by Order. The total number of genomes examined for each order is in parentheses.
(TIFF)

Figure S3 **Culture experiments to determine the concentrations of C1 and methylated compounds for ATP and radioisotope assays.** HTCC1062 cells were cultured in seawater medium amended with 10 µM NH_4_Cl, 1 µM KH_2_PO_4_, 10 nM FeCl_3_, vitamins, and C1 and methylated compounds at different concentrations.
(PDF)

Table S1 **Methylovory pathways and associated genes in** ***Candidatus*** **Pelagibacter ubique SAR11 HTCC1062.** Table includes genes involved in methylovory in SAR11 HTCC1062 and their associated homologs in other organisms mentioned in the text of the paper. Associated proteins are grouped together. Identifiers and annotated functions are taken from the Integrated Microbial Genomes database (IMG) where available, and from the literature where marked. Clusters of Orthologous Groups assignments (COGS) were taken from the IMG database, where available, and were generated using the COGnitor tool (available from http://www.ncbi.nlm.nih.gov/COG/) for sequences referred to the literature sources. Locus names are taken from IMG where available.
(XLS)

Table S2 **The percent abundance of SAR11 C1 metabolism genes at each sample site in GOS data, relative to** ***recA.***
(XLS)

Table S3 **Accession numbers used in** **Figure 3** **and** **Figure 4** **.**
(DOC)

## References

[1] Henderson JF (1979). Teaching one-carbon metabolism.. Biochem Educ.

[2] McDowell LR (2000). Vitamins in animal and human nutrition: Iowa State University Press.

[3] Chistoserdova L, Vorholt JA, Thauer RK, Lidstrom ME (1998). C1 transfer enzymes and coenzymes linking methylotrophic bacteria and methanogenic Archaea.. Science.

[4] Chistoserdova L, Chen SW, Lapidus A, Lidstrom ME (2003). Methylotrophy in *Methylobacterium extorquens* AM1 from a genomic point of view.. J Bacteriol.

[5] Chistoserdova L, Kalyuzhnaya MG, Lidstrom ME (2009). The expanding world of methylotrophic metabolism.. Annu Rev Microbiol.

[6] Giovannoni SJ, Hayakawa DH, Tripp HJ, Stingl U, Givan SA (2008). The small genome of an abundant coastal ocean methylotroph.. Environ Microbiol.

[7] Vorholt JA (2002). Cofactor-dependent pathways of formaldehyde oxidation in methylotrophic bacteria.. Arch Microbiol.

[8] Bicknell B, Owens JD (1980). Utilization of methylamines as nitrogen sources by non-methylotrophs.. J Gen Microbiol.

[9] Diaz MR, Visscher PT, Taylor BF (1992). Metabolism of dimethylsulfoniopropionate and glycine betaine by a marine bacterium.. FEMS Microbiol Lett.

[10] Barrett EL, Kwan HS (1985). Bacterial reduction of trimethylamine oxide.. Annu Rev of Microbiol.

[11] Stefels J (2000). Physiological aspects of the production and conversion of DMSP in marine algae and higher plants.. J Sea Res.

[12] Heikes BG, Chang W, Pilson MEQ, Swift E, Singh HB (2002). Atmospheric methanol budget and ocean implication.. Global Biogeochem Cycles.

[13] Singh HB, Tabazadeh A, Evans MJ, Field BD, Jacob DJ (2003). Oxygenated volatile organic chemicals in the oceans: Inferences and implications based on atmospheric observations and air-sea exchange models.. Geophys Res Lett.

[14] Dixon JL, Beale R, Nightingale PD (2010). Microbial methanol uptake in northeast Atlantic waters.. ISME J.

[15] Zimmerman PR, Chatfield RB, Fishman J, Crutzen PJ, Hanst PL (1978). Estimates on the production of CO and H_2_ from the oxidation of hydrocarbon emissions from vegetation.. Geophys Res Lett.

[16] Goode JG, Yokelson RJ, Ward DE, Susott RA, Babbitt RE (2000). Measurements of excess O_3_, CO_2_, CO, CH_4_, C_2_H_4_, C_2_H_2_, HCN, NO, NH_3_, HCOOH, CH_3_COOH, HCHO, and CH_3_OH in 1997 Alaskan biomass burning plumes by airborne Fourier transform infrared spectroscopy (AFTIR).. J Geophys Res.

[17] Mopper K, Zhou X, Kieber RJ, Kieber DJ, Sikorski RJ (1991). Photochemical degradation of dissolved organic carbon and its impact on the oceanic carbon cycle.. Nature.

[18] Jones DP, Thor H, Andersson B, Orrenius S (1978). Detoxification reactions in isolated hepatocytes. Role of glutathione peroxidase, catalase, and formaldehyde dehydrogenase in reactions relating to N-demethylation by the cytochrome P-450 system.. J Biol Chem.

[19] Vorholt JA, Chistoserdova L, Stolyar SM, Thauer RK, Lidstrom ME (1999). Distribution of tetrahydromethanopterin-dependent enzymes in methylotrophic bacteria and phylogeny of methenyl tetrahydromethanopterin cyclohydrolases.. J Bacteriol.

[20] Studer A, McAnulla C, Buchele R, Leisinger T, Vuilleumier S (2002). Chloromethane-induced genes define a third C1 utilization pathway in *Methylobacterium chloromethanicum* CM4.. J Bacteriol.

[21] Wilson SM, Gleisten MP, Donohue TJ (2008). Identification of proteins involved in formaldehyde metabolism by *Rhodobacter sphaeroides*.. Microbiology.

[22] Meskys R, Harris RJ, Casaite V, Basran J, Scrutton NS (2001). Organization of the genes involved in dimethylglycine and sarcosine degradation in *Arthrobacter* spp.: implications for glycine betaine catabolism.. Eur J Biochem.

[23] Grafstrom RC, Fornace AJ, Autrup H, Lechner JF, Harris CC (1983). Formaldehyde damage to DNA and inhibition of DNA repair in human bronchial cells.. Science.

[24] Craft TR, Bermudez E, Skopek TR (1987). Formaldehyde mutagenesis and formation of DNA-protein crosslinks in human lymphoblasts in vitro.. Mutat Res.

[25] Morris RM, Rappe MS, Connon SA, Vergin KL, Siebold WA (2002). SAR11 clade dominates ocean surface bacterioplankton communities.. Nature.

[26] Rappe MS, Giovannoni SJ (2003). The uncultured microbial majority.. Annu Rev Microbiol.

[27] Giovannoni SJ, Britschgi TB, Moyer CL, Field KG (1990). Genetic diversity in Sargasso Sea bacterioplankton.. Nature.

[28] Giovannoni SJ, Tripp HJ, Givan S, Podar M, Vergin KL (2005). Genome streamlining in a cosmopolitan oceanic bacterium.. Science.

[29] Carlson CA, Morris R, Parsons R, Treusch AH, Giovannoni SJ (2009). Seasonal dynamics of SAR11 populations in the euphotic and mesopelagic zones of the northwestern Sargasso Sea.. ISME J.

[30] Chistoserdova L, Laukel M, Portais JC, Vorholt JA, Lidstrom ME (2004). Multiple formate dehydrogenase enzymes in the facultative methylotroph *Methylobacterium extorquens* AM1 are dispensable for growth on methanol.. J Bacteriol.

[31] Chistoserdova L, Crowther GJ, Vorholt JA, Skovran E, Portais JC (2007). Identification of a fourth formate dehydrogenase in *Methylobacterium extorquens* AM1 and confirmation of the essential role of formate oxidation in methylotrophy.. J Bacteriol.

[32] Guse A, Stevenson CE, Kuper J, Buchanan G, Schwarz G (2003). Biochemical and structural analysis of the molybdenum cofactor biosynthesis protein MobA.. J Biol Chem.

[33] Wang W, Zhang W, Lu J, Yang Y, Chiao J (2002). MoeA, an enzyme in the molybdopterin synthesis pathway, is required for rifamycin SV production in *Amycolatopsis mediterranei* U32.. Appl Microbiol Biotechnol.

[34] Hasona A, Ray RM, Shanmugam KT (1998). Physiological and genetic analyses leading to identification of a biochemical role for the *moeA* (molybdate metabolism) gene product in *Escherichia coli*.. J Bacteriol.

[35] Robbertse B, Yoder RJ, Boyd A, Reeves J, Spatafora JW (2011). Hal: an automated pipeline for phylogenetic analyses of genomic data.. PLoS Curr 3: RRN1213.

[36] Chen Y, McAleer KL, Murrell JC (2010). Monomethylamine as a nitrogen source for a nonmethylotrophic bacterium, *Agrobacterium tumefaciens*.. Appl Environ Microbiol.

[37] Manian SS, Gumbleton R, Buckley AM, O'Gara F (1984). Nitrogen fixation and carbon dioxide assimilation in *Rhizobium japonicum*.. Appl Environ Microbiol.

[38] Singh RK, Singh RM (1983). Diverse effects of formate on the assimilatory metabolism of nitrate and nitrite in *Rhizobium*.. J Biosciences.

[39] de Vries GE, Arfman N, Terpstra P, Dijkhuizen L (1992). Cloning, expression, and sequence analysis of the *Bacillus methanolicus* C1 methanol dehydrogenase gene.. J Bacteriol.

[40] Liu X, Dong Y, Zhang J, Zhang A, Wang L (2009). Two novel metal-independent long-chain alkyl alcohol dehydrogenases from *Geobacillus thermodenitrificans* NG80-2.. Microbiology.

[41] Antoine E, Rolland JL, Raffin JP, Dietrich J (1999). Cloning and over-expression in *Escherichia coli* of the gene encoding NADPH group III alcohol dehydrogenase from *Thermococcus hydrothermalis*. Characterization and comparison of the native and the recombinant enzymes.. Eur J Biochem.

[42] Bystrykh LV, Arfman N, Dijkhuizen L, Murrell, JC, Kelly, DP (1993). The methanol-oxidizing enzyme systems in Gram-positive methylotrophic bacteria..

[43] Arfman N, Hektor HJ, Bystrykh LV, Govorukhina NI, Dijkhuizen L (1997). Properties of an NAD(H)-containing methanol dehydrogenase and its activator protein from *Bacillus methanolicus*.. Eur J Biochem.

[44] Vandecasteele J-P (2008). Petroleum microbiology: concepts environmental implications industrial applications..

[45] Serra AL, Mariscotti JF, Barra JL, Lucchesi GI, Domenech CE (2002). Glycine betaine transmethylase mutant of *Pseudomonas aeruginosa*.. J Bacteriol.

[46] Barra L, Fontenelle C, Ermel G, Trautwetter A, Walker GC (2006). Interrelations between glycine betaine catabolism and methionine biosynthesis in *Sinorhizobium meliloti* strain 102F34.. J Bacteriol.

[47] Kiene RP (1998). Uptake of choline and its conversion to glycine betaine by bacteria in estuarine waters.. Appl Environ Microbiol.

[48] Latypova E, Yang S, Wang YS, Wang T, Chavkin TA (2010). Genetics of the glutamate-mediated methylamine utilization pathway in the facultative methylotrophic beta-proteobacterium *Methyloversatilis universalis* FAM5.. Mol Microbiol.

[49] Chlumsky LJ, Zhang L, Jorns MS (1995). Sequence analysis of sarcosine oxidase and nearby genes reveals homologies with key enzymes of folate one-carbon metabolism.. J Biol Chem.

[50] Scott DA, Hickerson SM, Vickers TJ, Beverley SM (2008). The role of the mitochondrial glycine cleavage complex in the metabolism and virulence of the protozoan parasite *Leishmania major*.. J Biol Chem.

[51] Dworkin M, Falkow S, Rosenberg E, Schleifer K-H, Stackebrandt E (2006). The prokaryotes: Symbiotic associations, biotechnology, applied microbiology..

[52] Howard EC, Henriksen JR, Buchan A, Reisch CR, Burgmann H (2006). Bacterial taxa that limit sulfur flux from the ocean.. Science.

[53] Reisch CR, Moran MA, Whitman WB (2008). Dimethylsulfoniopropionate-dependent demethylase (DmdA) from *Pelagibacter ubique* and *Silicibacter pomeroyi*.. J Bacteriol.

[54] Teplyakov A, Obmolova G, Sarikaya E, Pullalarevu S, Krajewski W (2004). Crystal structure of the YgfZ protein from *Escherichia coli* suggests a folate-dependent regulatory role in one-carbon metabolism.. J Bacteriol.

[55] Scrutton NS, Leys D (2005). Crystal structure of DMGO provides a prototype for a new tetrahydrofolate-binding fold.. Biochem Soc Trans.

[56] Lin CN, Syu WJ, Sun WS, Chen JW, Chen TH (2010). A role of *ygfZ* in the *Escherichia coli* response to plumbagin challenge.. J Biomed Sci.

[57] Goenrich M, Bartoschek S, Hagemeier CH, Griesinger C, Vorholt JA (2002). A glutathione-dependent formaldehyde-activating enzyme (Gfa) from *Paracoccus denitrificans* detected and purified via two-dimensional proton exchange NMR spectroscopy.. J Biol Chem.

[58] Ras J, Van Ophem PW, Reijnders WN, Van Spanning RJ, Duine JA (1995). Isolation, sequencing, and mutagenesis of the gene encoding NAD- and glutathione-dependent formaldehyde dehydrogenase (GD-FALDH) from *Paracoccus denitrificans*, in which GD-FALDH is essential for methylotrophic growth.. J Bacteriol.

[59] Harms N, Ras J, Reijnders WN, van Spanning RJ, Stouthamer AH (1996). S-formylglutathione hydrolase of *Paracoccus denitrificans* is homologous to human esterase D: a universal pathway for formaldehyde detoxification?. J Bacteriol.

[60] Karl DM, Beversdorf L, Bjorkman KM, Church MJ, Martinez A (2008). Aerobic production of methane in the sea.. Nature Geosci.

[61] Tripp HJ, Schwalbach MS, Meyer MM, Kitner JB, Breaker RR (2009). Unique glycine-activated riboswitch linked to glycine-serine auxotrophy in SAR11.. Environ Microbiol.

[62] Schwalbach MS, Tripp HJ, Steindler L, Smith DP, Giovannoni SJ (2009). The presence of the glycolysis operon in SAR11 genomes is positively correlated with ocean productivity.. Environ Microbiol.

[63] Ben-Bassat A, Goldberg I, Mateles RI (1980). Distribution of methanol carbon between assimilation and oxidation pathways in methanol-grown *Pseudomonas* C.. J Gen Microbiol.

[64] Tripp HJ, Kitner JB, Schwalbach MS, Dacey JW, Wilhelm LJ (2008). SAR11 marine bacteria require exogenous reduced sulphur for growth.. Nature.

[65] King GM, Weber CF (2007). Distribution, diversity and ecology of aerobic CO-oxidizing bacteria.. Nat Rev Microbiol.

[66] Sorokin DY, Tourova TP, Kovaleva OL, Kuenen JG, Muyzer G (2010). Aerobic carboxydotrophy under extremely haloalkaline conditions in *Alkalispirillum/Alkalilimnicola* strains isolated from soda lakes.. Microbiology.

[67] Thrash JC, Boyd A, Huggett MJ, Grote J, Carini P (2011). Phylogenomic evidence for a common ancestor of mitochondria and the SAR11 clade.. Sci Rep.

[68] Atteia A, Adrait A, Brugiere S, Tardif M, van Lis R (2009). A proteomic survey of *Chlamydomonas reinhardtii* mitochondria sheds new light on the metabolic plasticity of the organelle and on the nature of the alpha-proteobacterial mitochondrial ancestor.. Mol Biol Evol.

[69] Appling DR (1991). Compartmentation of folate-mediated one-carbon metabolism in eukaryotes.. FASEB J.

[70] Pike ST, Rajendra R, Artzt K, Appling DR (2009). Mitochondrial C1-tetrahydrofolate synthase (MTHFD1L) supports the flow of mitochondrial one-carbon units into the methyl cycle in embryos.. J Biol Chem.

[71] Gabaldón T, Huynen MA (2007). From endosymbiont to host-controlled organelle: the hijacking of mitochondrial protein synthesis and metabolism.. PLoS Comput Biol.

[72] Finn RD, Mistry J, Tate J, Coggill P, Heger A (2010). The Pfam protein families database.. Nucleic Acids Res.

[73] Kanehisa M, Goto S, Furumichi M, Tanabe M, Hirakawa M (2010). KEGG for representation and analysis of molecular networks involving diseases and drugs.. Nucleic Acids Res.

[74] Dehal PS, Joachimiak MP, Price MN, Bates JT, Baumohl JK (2009). MicrobesOnline: an integrated portal for comparative and functional genomics.. Nucleic Acids Res.

[75] Edgar RC (2004). MUSCLE: a multiple sequence alignment method with reduced time and space complexity.. BMC Bioinformatics.

[76] Edgar RC (2004). MUSCLE: multiple sequence alignment with high accuracy and high throughput.. Nucleic Acids Res.

[77] Abascal F, Zardoya R, Posada D (2005). ProtTest: selection of best-fit models of protein evolution.. Bioinformatics.

[78] Stamatakis A (2006). RAxML-VI-HPC: maximum likelihood-based phylogenetic analyses with thousands of taxa and mixed models.. Bioinformatics.

[79] Robbertse B, Reeves JB, Schoch CL, Spatafora JW (2006). A phylogenomic analysis of the Ascomycota.. Fungal Genet Biol.

[80] van Dongen S (2000). Graph Clustering by Flow Simulation.. http://igitur-archive.library.uu.nl/dissertations/1895620/inhoud.htm.

[81] Wilhelm LJ, Tripp HJ, Givan SA, Smith DP, Giovannoni SJ (2007). Natural variation in SAR11 marine bacterioplankton genomes inferred from metagenomic data.. Biol Direct.

[82] Biers EJ, Sun S, Howard EC (2009). Prokaryotic genomes and diversity in surface ocean waters: interrogating the global ocean sampling metagenome.. Appl Environ Microbiol.

