# Supplementary figures and images for: One Carbon Metabolism in SAR11 Pelagic Marine Bacteria
Source: PLoS One. 2011 Aug 23;6(8):e23973. doi: 10.1371/journal.pone.0023973 (PMC3160333; doi:10.1371/journal.pone.0023973)

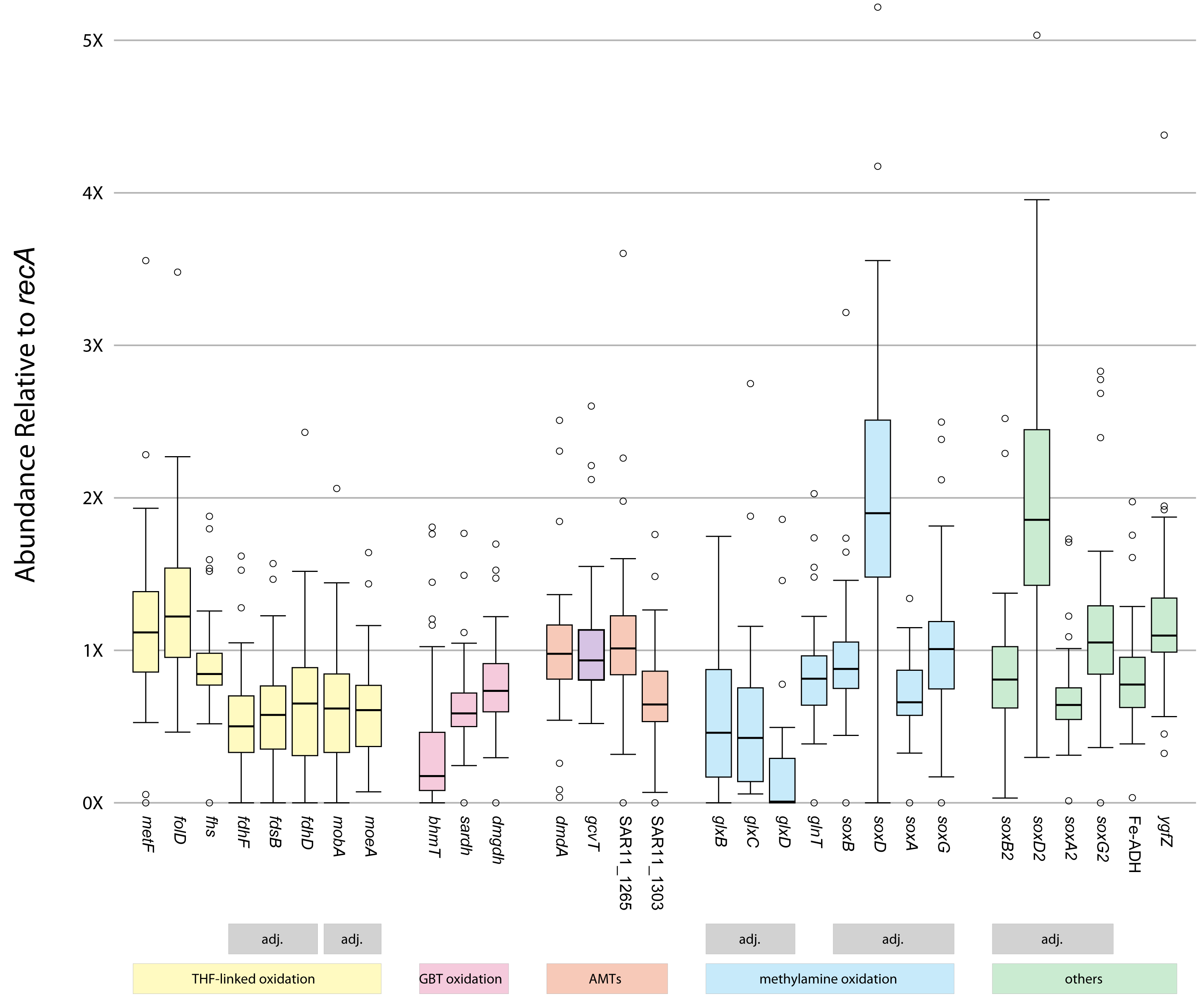

Supplement: Figure S1 — The abundance of SAR11 C1 metabolism genes in GOS data, relative to SAR11 recA genes. Genes were identified as SAR11 by a reciprocal best BLAST (RBB) approach. SAR11 C1 genes with frequencies less than SAR11 recA (<1x) may indicate that only subpopulations of SAR11 cells possess that gene; genes greater than 1x suggest that multiple copies of that gene are present per cell. Boxes encompass points between the 25th and 75th percentiles, with the median represented as a thick horizontal line. Whiskers span the minimal distance needed to include all points within 1.5 x the interquartile range beyond the interquartile boundary, with points outside of this range rendered individually as circles. For each gene, n = 40. Abbreviations: adj., genomically adjacent genes. (TIF) [file pone.0023973.s001.tif]

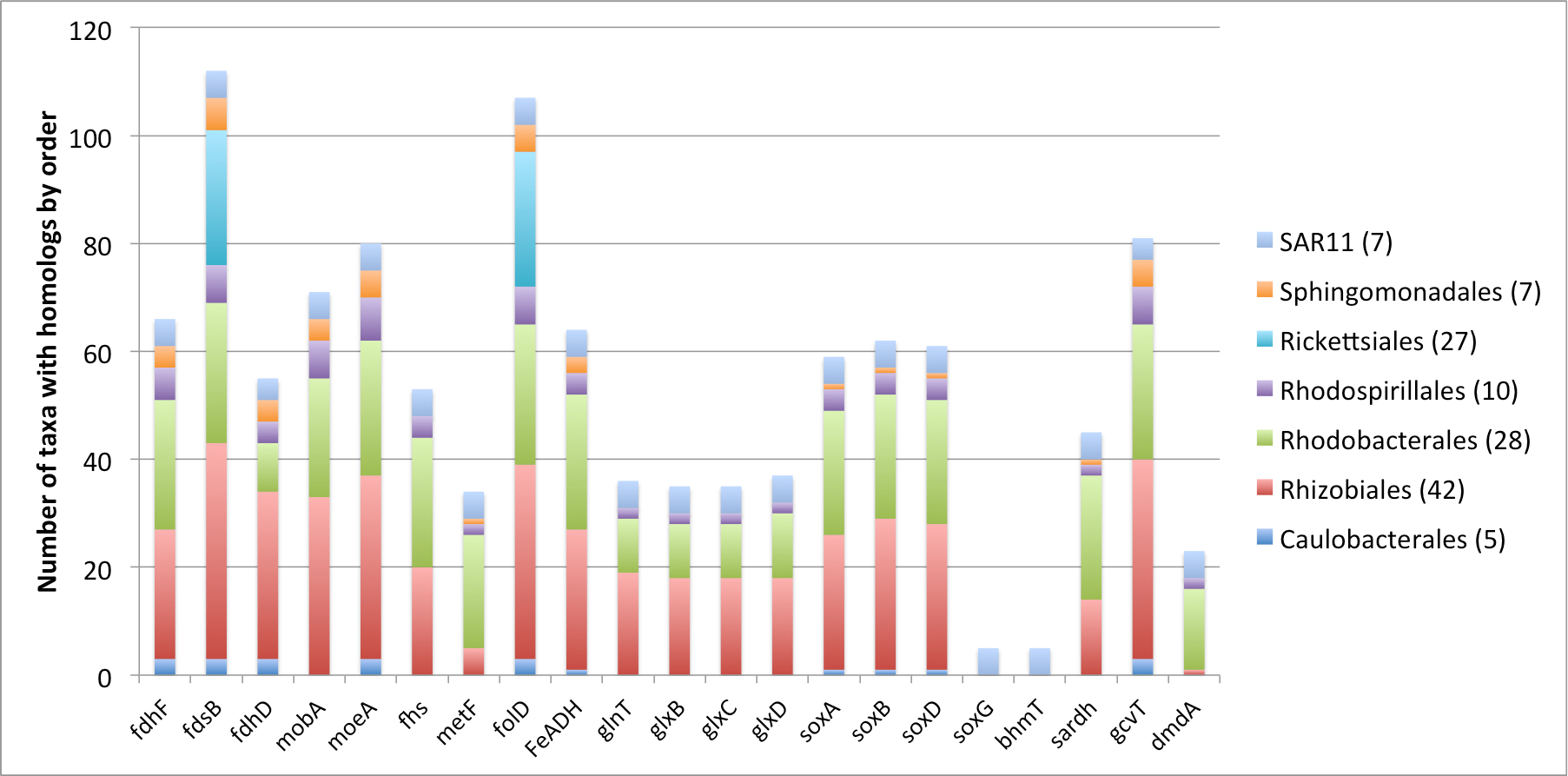

Supplement: Figure S2 — Distribution of C1 gene homologs throughout the Alphaproteobacteria . The number of genomes containing homologs of C1 oxidation genes reported by gene and divided by Order. The total number of genomes examined for each order is in parentheses. (TIFF) [file pone.0023973.s002.tiff]

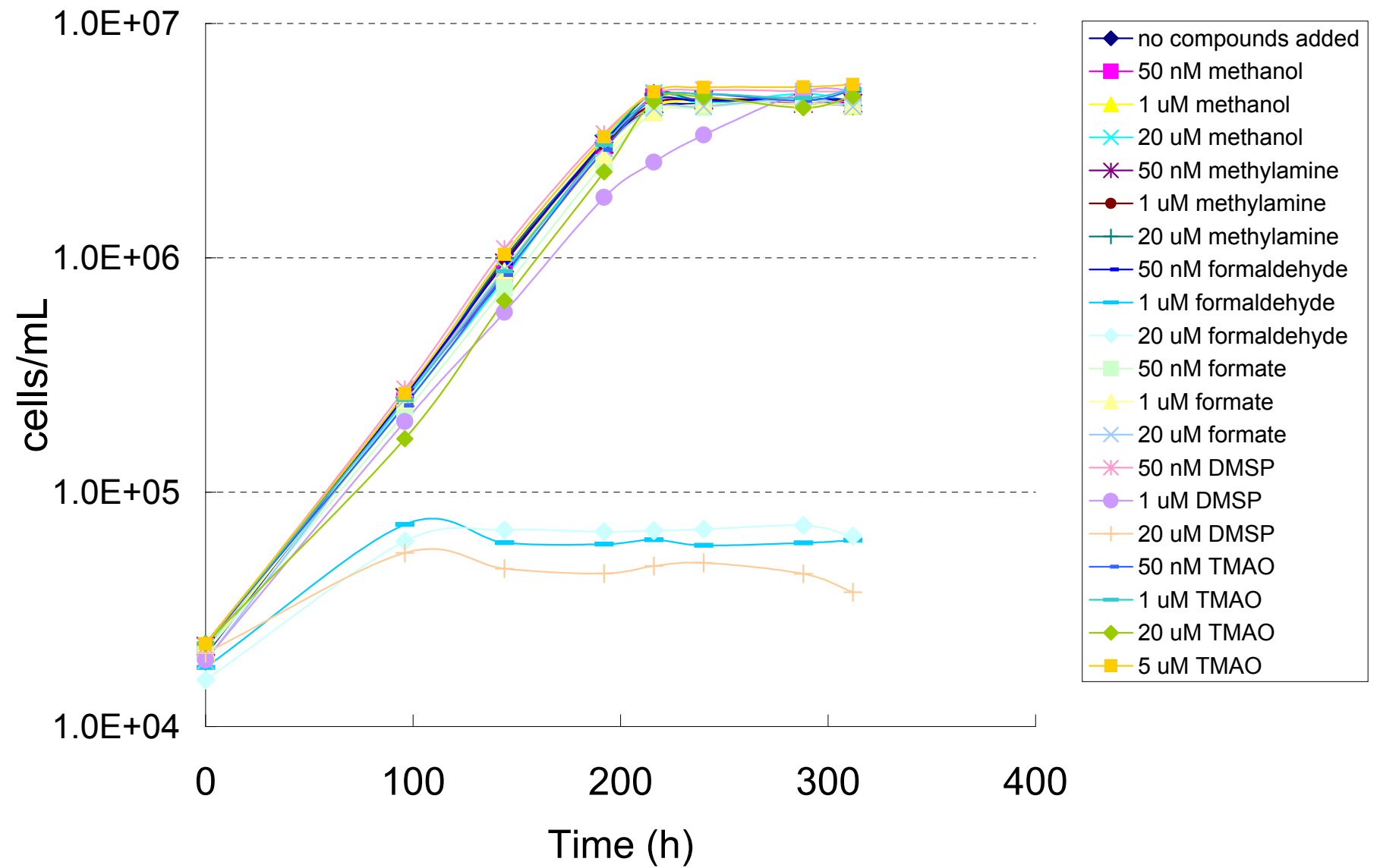

Supplement: Figure S3 — Culture experiments to determine the concentrations of C1 and methylated compounds for ATP and radioisotope assays. HTCC1062 cells were cultured in seawater medium amended with 10 µM NH4Cl, 1 µM KH2PO4, 10 nM FeCl3, vitamins, and C1 and methylated compounds at different concentrations. (PDF) [file pone.0023973.s003.pdf]
