# Supplementary material for: One Carbon Metabolism in SAR11 Pelagic Marine Bacteria
Source: PLoS One. 2011 Aug 23;6(8):e23973. doi: 10.1371/journal.pone.0023973 (PMC3160333; doi:10.1371/journal.pone.0023973)
Supplement: Table S3 — Accession numbers used in Figure 3 and Figure 4 . (DOC) [file pone.0023973.s006.doc]

**Table S3 Accession numbers used in Figure 3 and Figure 4.**

| Accession number | Annotation | Organism |
| --- | --- | --- |
| YP_002545013.1 | alcohol dehydrogenase protein | Agrobacterium radiobacter K84 |
| NP_355100.2 | dehydrogenase | Agrobacterium tumefaciens str. C58 |
| YP_002550133.1 | dehydrogenase | Agrobacterium vitis S4 |
| ZP_01447774.1 | alcohol dehydrogenase, iron-containing | alpha proteobacterium HTCC2255 |
| P31005 | RecName: Full=NAD-dependent methanol dehydrogenase; Short=MEDH | Bacillus methanolicus |
| YP_223278.1 | alcohol dehydrogenase, iron-containing | Brucella abortus bv. 1 str. 9-941 |
| ZP_05952403.1 | iron-containing alcohol dehydrogenase | Brucella pinnipedialis M163/99/10 |
| ZP_05182105.1 | 1,3-propanediol dehydrogenase | Brucella sp. 83/13 |
| ZP_05069281.1 | NAD-dependent 4-hydroxybutyrate dehydrogenase | Candidatus Pelagibacter sp. HTCC7211 |
| ZP_01264921.1 | iron-containing alcohol dehydrogenase | Candidatus Pelagibacter ubique HTCC1002 |
| YP_266695.1 | iron-containing alcohol dehydrogenase | Candidatus Pelagibacter ubique HTCC1062 |
| YP_572741.1 | iron-containing alcohol dehydrogenase | Chromohalobacter salexigens DSM 3043 |
| YP_001126968 | NADH-dependent butanol dehydrogenase A | Geobacillus thermodenitrificans NG80-2 |
| YP_437390.1 | alcohol dehydrogenase, class IV | Hahella chejuensis KCTC 2396 |
| ZP_02167095.1 | putative alcohol dehydrogenase protein | Hoeflea phototrophica DFL-43 |
| ZP_05115658.1 | alcohol dehydrogenase, iron-containing superfamily | Labrenzia alexandrii DFL-11 |
| ZP_05111270.1 | alcohol dehydrogenase, iron containing | Legionella drancourtii LLAP12 |
| CBJ11468.1 | putative alcohol dehydrogenase | Legionella longbeachae NSW150 |
| YP_001251001.1 | alcohol dehydrogenase | Legionella pneumophila str. Corby |
| ZP_01001968.1 | Iron-containing alcohol dehydrogenase | Loktanella vestfoldensis SKA53 |
| ZP_01625980.1 | Alcohol dehydrogenase, class IV | marine gamma proteobacterium HTCC2080 |
| ZP_01617418.1 | Alcohol dehydrogenase, class IV | marine gamma proteobacterium HTCC2143 |
| ZP_01074771.1 | hypothetical methanol dehydrogenase, NAD-dependent | Marinomonas sp. MED121 |
| NP_104263.1 | dehydrogenase, (iron-containing alcohol dehydrogenase, 4-hydroxybutyrate dehydrogenase, methanol dehydrogenase) | Mesorhizobium loti MAFF303099 |
| ZP_05808837.1 | iron-containing alcohol dehydrogenase | Mesorhizobium opportunistum WSM2075 |
| YP_674426.1 | iron-containing alcohol dehydrogenase | Mesorhizobium sp. BNC1 |
| ZP_01899799.1 | alcohol dehydrogenase, iron containing | Moritella sp. PE36 |
| ZP_01126276.1 | Alcohol dehydrogenase, class IV | Nitrococcus mobilis Nb-231 |
| ZP_02152523.1 | alcohol dehydrogenase, iron-containing | Oceanibulbus indolifex HEL-45 |
| ZP_00998327.1 | alcohol dehydrogenase, iron-containing | Oceanicola batsensis HTCC2597 |
| YP_001372417.1 | iron-containing alcohol dehydrogenase | Ochrobactrum anthropi ATCC 49188 |
| ZP_04682498.1 | 1,3-propanediol dehydrogenase | Ochrobactrum intermedium LMG 3301 |
| ZP_05052438.1 | alcohol dehydrogenase, iron-containing superfamily | Octadecabacter antarcticus 307 |
| YP_918308.1 | iron-containing alcohol dehydrogenase | Paracoccus denitrificans PD1222 |
| YP_001414057.1 | iron-containing alcohol dehydrogenase | Parvibaculum lavamentivorans DS-1 |
| ZP_02144351.1 | iron-containing alcohol dehydrogenase | Phaeobacter gallaeciensis BS107 |
| ZP_01222355.1 | hypothetical methanol dehydrogenase, NAD-dependent | Photobacterium profundum 3TCK |
| YP_132532.1 | methanol dehydrogenase | Photobacterium profundum SS9 |
| ZP_01906929.1 | Alcohol dehydrogenase, class IV | Plesiocystis pacifica SIR-1 |
| YP_001186178.1 | iron-containing alcohol dehydrogenase | Pseudomonas mendocina ymp |
| ZP_04585725.1 | methanol dehydrogenase, NAD-dependent | Pseudomonas syringae pv. oryzae str. 1_6 |
| YP_274618.1 | alcohol dehydrogenase, iron-containing | Pseudomonas syringae pv. phaseolicola 1448A |
| YP_235819.1 | Iron-containing alcohol dehydrogenase | Pseudomonas syringae pv. syringae B728a |
| NP_792754.1 | methanol dehydrogenase, NAD-dependent | Pseudomonas syringae pv. tomato str. DC3000 |
| ZP_05086175.1 | NAD-dependent 4-hydroxybutyrate dehydrogenase | Pseudovibrio sp. JE062 |
| ZP_01255682.1 | iron-containing alcohol dehydrogenase | Psychroflexus torquis ATCC 700755 |
| YP_001979163.1 | probable alcohol dehydrogenase protein | Rhizobium etli CIAT 652 |
| YP_002826658.1 | putative dehydrogenase | Rhizobium sp. NGR234 |
| ZP_05843391.1 | iron-containing alcohol dehydrogenase | Rhodobacter sp. SW2 |
| YP_352372.1 | Iron-containing alcohol dehydrogenase | Rhodobacter sphaeroides 2.4.1 |
| YP_001167810.1 | iron-containing alcohol dehydrogenase | Rhodobacter sphaeroides ATCC 17025 |
| ZP_05125079.1 | iron-containing alcohol dehydrogenase | Rhodobacteraceae bacterium KLH11 |
| ZP_05073345.1 | iron-containing alcohol dehydrogenase | Rhodobacterales bacterium HTCC2083 |
| ZP_01741605.1 | alcohol dehydrogenase, iron-containing | Rhodobacterales bacterium HTCC2150 |
| YP_682452.1 | NAD-dependent 4-hydroxybutyrate dehydrogenase | Roseobacter denitrificans OCh 114 |
| ZP_01904301.1 | iron-containing alcohol dehydrogenase | Roseobacter sp. AzwK-3b |
| ZP_05102413.1 | iron-containing alcohol dehydrogenase | Roseobacter sp. GAI101 |
| ZP_01058795.1 | alcohol dehydrogenase, iron-containing | Roseobacter sp. MED193 |
| ZP_01755487.1 | alcohol dehydrogenase, iron-containing | Roseobacter sp. SK209-2-6 |
| ZP_00961489.1 | hypothetical protein ISM_11415 | Roseovarius nubinhibens ISM |
| ZP_01037832.1 | alcohol dehydrogenase, iron-containing | Roseovarius sp. 217 |
| ZP_01446139.1 | alcohol dehydrogenase, iron-containing | Roseovarius sp. HTCC2601 |
| ZP_01878451.1 | iron-containing alcohol dehydrogenase | Roseovarius sp. TM1035 |
| YP_167820.1 | alcohol dehydrogenase, iron-containing | Ruegeria pomeroyi DSS-3 |
| ZP_05087994.1 | iron-containing alcohol dehydrogenase | Ruegeria sp. R11 |
| ZP_01744995.1 | alcohol dehydrogenase, iron-containing | Sagittula stellata E-37 |
| YP_001676072.1 | iron-containing alcohol dehydrogenase | Shewanella halifaxensis HAW-EB4 |
| YP_002309841.1 | Methanol dehydrogenase, NAD-dependent, putative | Shewanella piezotolerans WP3 |
| YP_001474265.1 | alcohol dehydrogenase | Shewanella sediminis HAW-EB3 |
| YP_001760482.1 | iron-containing alcohol dehydrogenase | Shewanella woodyi ATCC 51908 |
| ZP_05784705.1 | iron-containing alcohol dehydrogenase | Silicibacter lacuscaerulensis ITI-1157 |
| ZP_05741575.1 | iron-containing alcohol dehydrogenase | Silicibacter sp. TrichCH4B |
| YP_001327796.1 | iron-containing alcohol dehydrogenase | Sinorhizobium medicae WSM419 |
| ZP_01549185.1 | dehydrogenase, (iron-containing alcohol dehydrogenase, 4-hydroxybutyrate dehydrogenase, methanol dehydrogenase) | Stappia aggregata IAM 12614 |
| ZP_00948444.1 | alcohol dehydrogenase, iron-containing | Sulfitobacter sp. NAS-14.1 |
| ZP_05341556.1 | NAD-dependent 4-hydroxybutyrate dehydrogenase | Thalassiobium sp. R2A62 |
| CAA74334 | alcohol dehydrogenase | Thermococcus hydrothermalis |
| ACB13616.1 | NAD-dependent 4-hydroxybutyrate dehydrogenase | uncultured alpha proteobacterium 01-003886 |
| ABZ05924.1 | putative iron-containing alcohol dehydrogenase | uncultured marine microorganism HF4000_001B09 |
| ABZ05955.1 | putative iron-containing alcohol dehydrogenase | uncultured marine microorganism HF4000_001L24 |
| YP_544550.1 | aminomethyltransferase | Methylobacillus flagellatus KT |
| [YP_001533657.1](http://www.ncbi.nlm.nih.gov/entrez/viewer.fcgi?db=protein&id=YP_001533657.1). | aminomethyltransferase | [Dinoroseobacter shibae (strain DFL 12)](http://www.uniprot.org/taxonomy/398580) |
| [YP_643544.1](http://www.ncbi.nlm.nih.gov/entrez/viewer.fcgi?db=protein&id=YP_643544.1). | aminomethyltransferase | [Rubrobacter xylanophilus (strain DSM 9941 / NBRC 16129)](http://www.uniprot.org/taxonomy/266117) |
| NC_006395 | aminomethyltransferase | Haloarcula marismortui ATCC 43049 |
| [ZP_05913927.1](http://www.ncbi.nlm.nih.gov/protein/260905605?report=genbank&log$=protalign&blast_rank=1&RID=PV0N0R7K016) | putative aminomethyltransferase protein | Brevibacterium linens BL2 |
| [ZP_00959021.1](http://www.ncbi.nlm.nih.gov/protein/83950288?report=genbank&log$=protalign&blast_rank=1&RID=PV0WKAHG01S) | putative aminomethyltransferase protein | Roseovarius nubinhibens ISM |
| YP_001418071.1 | glycine cleavage T protein | Xanthobacter autotrophicus py2 |
| YP_745645.1 | aminomethyltransferase family protein | Granulibacter bethesdensis CGDNIH1 |
| YP_001524291.1 | aminomethyltransferase | Azorhizobium caulinodans ORS 571 |
| YP_952043.1 | glycine cleavage T protein (aminomethyl transferase) | Mycobacterium vanbaalenii PYR-1 |
| YP_590345.1 | glycine cleavage T protein | Acidobacteria bacterium Ellin345 (Candidatus Koribacter versatilis Ellin345) |
| NP_436414.1 | aminomethyltransferase | Sinorhizobium melloti 1021 |
| ZP_01034757.1 | aminomethyltransferase | Roseovarius sp.217 |
| YP_001412229.1 | glycine cleavage T protein (aminomethyl transferase) | Parvibaculum  lavamentivorans DS-1 |
| [YP_916134.1](http://www.ncbi.nlm.nih.gov/protein/119385078?report=genbank&log$=protalign&blast_rank=1&RID=PV1YUU1W016) | glycine cleavage system T protein | Paracoccus denitrificans PD1222 |
| [ZP_01444337.1](http://www.ncbi.nlm.nih.gov/protein/114765193?report=genbank&log$=protalign&blast_rank=1&RID=PV20PA3U016) | probable aminomethyltransferase (glycine cleavage system t protein) | Roseovarius sp. HTCC2601 (Pelagibaca bermudensis HTCC2601) |
| [ZP_01000988.1](http://www.ncbi.nlm.nih.gov/protein/84502875?report=genbank&log$=protalign&blast_rank=1&RID=PV22VWDV014) | hypothetical protein OB2597_14831 | Oceanicola batsensis HTCC2597 |
| NP_385210.1 | hypothetical protein SMc02558 | Sinorhizobium meliloti 1021 |
| [YP_001602561.1](http://www.ncbi.nlm.nih.gov/protein/162148100?report=genbank&log$=protalign&blast_rank=1&RID=PV2DVBA3011) | glycine cleavage system aminomethyltransferase T | Gluconacetobacter diazotrophicus PAl 5 |
| [NP_772391.1](http://www.ncbi.nlm.nih.gov/protein/27380862?report=genbank&log$=protalign&blast_rank=1&RID=PV2CWU6G01S) | glycine cleavage system aminomethyltransferase T | Bradyrhizobium japonicum USDA 110 |
| YP_001638331.1 | glycine cleavage system T protein | Methylobacterium extorquens PA1 |
| [YP_001210080.1](http://www.ncbi.nlm.nih.gov/protein/146329402?report=genbank&log$=protalign&blast_rank=1&RID=PV2J6T8201S) | glycine cleavage system T protein | Dichelobacter nodosus VCS1703A |
| YP_001609202.1 | aminomethyltransferase | Bartonella tribocorum CIP 105476 |
| [YP_001257682.1](http://www.ncbi.nlm.nih.gov/protein/148558325?report=genbank&log$=protalign&blast_rank=1&RID=PV3537HP01S) | glycine cleavage system aminomethyltransferase T | Brucella ovis ATCC 25840 |
| YP_680429.1 | aminomethyltransferase, putative | Roseobacter denitrificans OCh 114 |
